# Automated identification of honey bee pollen loads for field‐applied palynological studies

**DOI:** 10.1111/nph.70435

**Published:** 2025-08-02

**Authors:** Jonathan Barés, Pascal Poncelet, Christine M. Doucet, Charline Legrand, Anais Cambon, Capucine Carlier, Patrick Chevin, Leo‐Paul Dewaele, Delphine Jullien, Jean‐Baptiste Thibaud, Pierre Charnet, Matthieu Rousset, Pierre‐Olivier Cheptou

**Affiliations:** ^1^ Laboratoire de Mécanique et Génie Civil, UMR 5508 CNRS Montpellier University 34095 Montpellier France; ^2^ Montpellier University, LIRMM, UMR 5506 34095 Montpellier France; ^3^ CBS (Centre de Biologie Structurale), Montpellier University, CNRS, INSERM 34293 Montpellier France; ^4^ CEFE UMR 5175, CNRS, Université de Montpellier, Université Paul‐Valery Montpellier, EPHE 34293 Montpellier Cedex 05 France; ^5^ IBMM UMR5247, CNRS, ENSCM, Université de Montpellier 34293 Montpellier France

**Keywords:** flowering phenology, honeybee, machine learning, pollen, species identification, UV/Vis spectroscopy

## Abstract

- In a changing world, it is crucial to characterise communities and their evolution over time. Because social insect pollinators forage on flowering plants around the colony, the nest potentially contains important information about the pollinated plants such as species identity and plant phenology. In this paper, we introduce new approaches to assess plant composition in a Mediterranean summer plant community from pollen foraged by honeybees.
- We leveraged the autofluorescence properties of the pollen load to classify plant species, both using a UV/Vis spectrophotometer in the laboratory and a dedicated prototype ‘pollen analyser’ adapted to field studies.
- Our results demonstrate that data collected from fluorescent spectra and pollen analyser measurements of pollen load from 14 plant species are specific enough to distinguish plant species. When combined with machine learning techniques, particularly the Support Vector Machine classifier, these approaches provide powerful methods to automatically identify species from fluorescence measurements of pollen load.
- Overall, our study shows that analysing the autofluorescence of honeybee pollen load enables the precise identification of their floral origins, paving the way for a real‐time, spatially distributed observatory of flowering plants to monitor species identity, flowering phenology and long‐term ecological dynamics.

## Introduction

In the context of global change and mass extinction, assessing plant composition in real time and space provides valuable insights into plant adaptation, plant community dynamics or plant‐pollinator network. Following flowering phenology of plant communities by identifying and quantifying pollen production in real time is a promising way to study plant communities. Interestingly, evolutionary ecology theory provides a rationale to relate pollen harvesting to flowering phenology. According to the ‘optimal foraging theory’, we expect that pollinators forage when the pollen resource of a given species is abundant, that is during the flowering peak of the plant species (Pyke *et al*., 1977; Stephens, 2008). Pollen collected by social insects and brought back to the nest potentially contains important information about the pollinated plants such as species identity, species abundance and plant phenology (Galimberti *et al*., 2014).

Honeybee (Kirk, 2022) *Apis mellifera*, now present in all continents except Antarctica, is a super‐generalist pollinator and the most frequent floral visitor in natural habitats and crops world‐wide (Hung *et al*., 2018). Among the 87.5% of plants pollinated by animals, at least 51% of the plant species are visited by *A. mellifera* (Valido *et al*., 2019). Therefore, monitoring plant communities visited by honeybees potentially represents a large panel of the overall community. Honeybee colonies, for which pollen is the primary source of protein, serve as valuable natural collectors of pollen samples in all ecosystems. Thus, analysing pollen harvested provides the founding principles to set up an observatory of entomophilous plants based on the analysis of the pollen load (corbicular pollen), that is, pollen harvested by honeybee foragers. Moreover, analysing pollen loads on bees may allow the development of a non‐invasive method.

During their collecting trips, forager bees pack hundreds to thousands of pollen grains from flowers with nectar and salivary substances to form a pollen load on their hind legs with the help of specialised combs and hairs (Matherne *et al*., 2021). A fresh pollen load typically ranges in size from 1.4 to 4 mm, weighs between 8 and 15 mg, and varies in colour from white to black, depending on the ratios of plant pigments and chemical composition, including phenolics or several elements like Calcium (Ca), Magnesium (Mg) or iron (Fe) (Bleha *et al*., 2019; Thakur & Nanda, 2020). One of the key characteristics of honeybee foraging behaviour is flower constancy, which, in theory, results in monospecific pollen loads (Grant, 1950; Campos *et al*., 1997; Hill *et al*., 1997; Grüter *et al*., 2011). However, foragers might also gather polyfloral pollen loads by harvesting pollen grains from several flower sources, but the ratio of polyfloral pollen load remains very limited, constituting < 2% of the total pollen load analysed in several studies (Pernal & Currie, 2001).

Palynology encompasses the analysis of pollen grains to establish taxonomic relationships, enabling the classification of collected samples and the identification of plant species within a given system. Pollen images are classically obtained through different types of microscopy, including light microscopy, electron microscopy and spectroscopic imaging, each providing complementary insights into the structural and compositional characteristics of pollen grains (Mander & Punyasena, 2014; Korinth *et al*., 2020; Prdun *et al*., 2021). Over the past two decades, the progress of machine learning approaches allowing the computational analysis of these palynological data led to automation of pollen counting and identification (Holt & Bennett, 2014). While automation enhances efficiency and accelerates these analyses, they still demand analytical expertise, hands‐on experimental tasks such as sample collection and preparation, and often rely on bulky equipment restricted to laboratory settings. Moreover, most pollen analysis systems have been developed to identify airborne pollen, resulting in the over‐representation of allergic anemophilous pollen in datasets and libraries (e.g. https://data.oreme.org/palyno/palyno_gallery). In addition, pollen identification by DNA analysis has been recently developed and successfully applied to determine the floral composition and geographic origin of honey (Hawkins *et al*., 2015; Wirta *et al*., 2021).

This taxonomic fidelity of foragers and the stereotypical species‐specific morphology observed at the pollen grain level (Topitzhofer *et al*., 2021) suggests the existence of corresponding species‐specific traits at the pollen load level. The most prominent pollen load species‐specific trait is their colour, which has already been used to identify the plant source of the pollen load (Kirk & International Bee Research Association, 2006; Kirk, 2018; Topitzhofer *et al*., 2021; Bailey *et al*., 2023). Colour analysis is usually made after manual extraction of the pollen load or using a dedicated pollen trap placed at the entrance of the hive. This approach has been successfully applied to studying honeybee diet (Colwell *et al*., 2017; Topitzhofer *et al*., 2019). An automated system based on computer vision and machine learning has been proposed to automatically authenticate the pollen origin from the pollen load colour analysis (Chica & Campoy, 2012, 2015; Brodschneider *et al*., 2021; Borlinghaus *et al*., 2023). However, many pollen loads from taxonomically distant plant species have hues so similar that they cannot be distinguished (Chica & Campoy, 2015). Using ultraviolet/high‐energy violet light helps the differentiation of loads of the same shade (Bailey *et al*., 2023). Conversely, Bleha *et al*. (2021) demonstrated that three samples of the same species (*Fagopyrum esculentum*) produce pollen loads of different tones (Bleha *et al*., 2021). These colour variations may result from genetic differences within species, as well as environmental factors such as humidity, soil properties, fungal presence and more (Modro *et al*., 2009). Finally, the quantification of the plant sources in the foraging area is usually estimated by weighing pollen loads after sorting them by colour, which raises reliability concerns. These include the over‐represented large‐sized pollen loads when using pollen traps, weight variations of pollen loads depending on the plant source or the potential omission of rare pollen colours when sampling is conducted (García‐García *et al*., 2004; Conti *et al*., 2016). While colour‐based identification provides important information, it must be complemented by other approaches.

When excited by UV or visible wavelengths radiation, intact living tissue emits natural radiation. This phenomenon, called autofluorescence, is due to the deactivation of natural fluorophores, and can be measured and quantified (Roshchina, 2012; García‐Plazaola *et al*., 2015). Microscopic and macroscopic fluorescence imaging techniques have been widely used to study the morphology and physiology of plants or to monitor their health status (Buschmann *et al*., 2001; Talamond *et al*., 2015). In particular, the strong auto‐fluorescence of pollen grains has been successfully exploited for taxonomic plant identification or quantification of pollen grains (Driessen *et al*., 1989; Aronne *et al*., 2001; Fonseca *et al*., 2002; Ronneberger *et al*., 2002; Castro *et al*., 2010; Punyasena *et al*., 2012; Johnsrud *et al*., 2013; Gabriele *et al*., 2015; Bailey *et al*., 2023). Mitsumoto *et al*. (2009) demonstrated that the combination of size and ratio of blue and red auto‐fluorescence of pollen grains can be used to identify plant species (Mitsumoto *et al*., 2009). Moreover, principal component analysis of the spectral data shows that pollen grains can be discriminated by comparing their spectral peak (Mitsumoto *et al*., 2009; Pan *et al*., 2011) or intensity (O'Connor *et al*., 2011, 2014) with high taxonomic accuracy. Auto‐fluorescence is therefore a promising methodology, and this work explores the possibility of transposing the floral identification of pollen grains based on spectral signatures to the pollen load level. Indeed, studying auto‐fluorescence at the pollen load level allows identifying species in a non‐invasive way. By studying 14 floral species from a Mediterranean summer plant community foraged by bees, we demonstrate that pollen loads can be automatically differentiated using a combination of fluorescent spectroscopy and machine learning techniques. Based on these results, we developed an innovative field‐adapted prototype, dedicated to identifying species of intact pollen loads.

## Materials and Methods

### The experimental apiary, pollen load collection and species

The experimental apiary, which typically contains between 5 and 20 hives, is located in the Labex CEMEB platform ‘terrains d'expériences’ in Montpellier (France), a 4‐ha area. The platform is located at the periphery of the city. The flora consists of common herbaceous species (e.g. *Lolium perenne* L. and *Thymus vulgaris* L.), trees (*Quercus ilex* L. and *Arbutus unedo* L.) and shrubs (*Phyllierea angustifolia* L. and *Salvi rosmarinus* L.) commonly found in French Mediterranean areas. In addition, the platform is surrounded by private gardens with ornamental species, which are potentially visited by the bees from our experimental hives. In June, in the Mediterranean area, various species are flowering simultaneously, and the weather is usually sunny and dry, which favours intense foraging activity by honeybee communities.

Forager honeybees with a visible pollen load were captured using a 50 ml Falcon® centrifuge tube on the flowers they were foraging on the experimental hive or closely around. Each Falcon tube was used for only one bee and for a single collection session. At least 10 forager bees have been captured for each flowering species. The bee‐containing falcon was placed for 10 min in a refrigerator at 4°C. The cold anesthetized bee was then picked up from the falcon and the two pollen loads were carefully collected with forceps. Forceps were cleaned between the samples. Following pollen load collection, honeybees were released. Each pollen load was then placed off into a well of a 24‐well plate and kept at room temperature in the dark. At the end of the collection, each pollen load was analysed (20 pollen loads per species or so), first using the pollen analyser and then using an ultra‐fast UV/vis spectrometer (see the ‘Manual light‐microscopy palynology’ and ‘Fluorescence spectroscopy on pollen load’ sections). A total of 14 pollen species were identified, and the analysis for each species was completed in under 4 h, for a total of 334 pollen loads. Plant species identification was carried out using the botanical expertise of the experimenter, with confirmation provided by the collaborative mobile application Pl@ntNet (https://plantnet.org/) (Neto‐Bradley *et al*., 2025) and manual light‐microscopy palynology. The 14 species collected and studied were: *Cirsium arvense* L. (Asteraceae), *Crepis foetida* L. (Asteraceae), *Diplotaxis erucoides* L. (Brassicaceae), *Echium vulgare* L. (Boraginaceae), *Knautia arvensis* L. (Caprifoliaceae), *Oenothera speciosa* L. (Onagraceae), *Prunus domestica* L. (Rosaceae), *Punica granatum* L. (Lythraceae), *Q. ilex* L. (Fagaceae), *Rhus coriaria* L. (Anacardiaceae), *Rubus fruticosus* L. (Rosaceae), *Rubus ulmifolius* L. (Rosaceae), *Scabiosa atropurpurea* L. (Caprifoliaceae) and *Vitis vinifera* L. (Vitaceae).

### Manual light‐microscopy palynology

Pollen identification was performed via light microscopy, enabling the correlation between specific plant taxa and fluorescence spectroscopy signals. Sixteen pollen loads from floral sources were randomly selected for analysis. Pollen grains were prepared through acetolysis following Erdtman's protocol (Erdtman, 1960). The acetolyzed pollen grains were mounted on glass slides using glycerol jelly as the mounting medium, sealed with coverslips and fixed with clear nail varnish to ensure long‐term preservation. Microscopic observations were carried out at ×400 to ×1000 magnification using a bright‐field microscope (Leica application suite v.4.13; Leica Microsystems (Switzerland) Ltd., Stereo and Macroscope Systems, Heerbrugg, Switzerland). Species identifications were achieved through morphological comparison with pollen reference databases by a trained palynologist. All analyses were conducted at the Institut des Sciences de l'Evolution de Montpellier (ISEM), University of Montpellier.

### Fluorescence spectroscopy on pollen load

Fresh pollen loads were placed in a black 384‐well flat‐bottom plate (#3575; Corning, Glendale, AZ, USA), one load per well. Emission spectra were collected on a ClarioSTAR (BMG Labtech, Ortenberg, Germany) equipped with Linear Variable Filters for excitation and emission. The focus was optimised on a single well. As loads may vary in size, the optimal focus was calculated at several well positions and the optimal position was determined and applied to the whole plate. The gain was adjusted on the whole plate for each excitation wavelength. Emission spectra were then collected for six excitation wavelengths (365, 390, 395, 460, 530 and 625 nm), with a resolution of 1 nm and bandwidths of 10 nm. The emission spectra began 50 nm after the excitation wavelength. The raw data were treated with custom Python scripts.

### The pollen analyser

The pollen analyser, which is dedicated to pollen identification, is a cylinder with internal dimensions of 34 mm in diameter and 21 mm in height, easy to use and portable. It is 3D printed with black (lower part) and grey (upper part) acrylonitrile butadiene styrene (ABS). The internal base of the lower part is covered with a small disposable white paper disc on which the pollen ball to be analysed is placed. The upper part of the box is sealed at the top with a 2‐mm‐thick glass pane measuring 2 cm by 2 cm, enabling a camera positioned 46 mm above the pane to capture clear images of the pollen balls inside. This camera is an 8‐megapixel Picam NoIR v.2.1 (Raspberry Pi Holdings, Cambridge, UK) equipped with an 8‐mm lens. The images are taken without an IR filter either in the camera or in the lens. Additionally, around the circumference of the upper part of the box, 12 evenly spaced holes accommodate LEDs positioned at a 45‐degree angle towards the centre of the box's lower section. These LEDs are arranged into four families of three, with each group oriented 120 degrees apart to provide uniform illumination of the pollen load. The first family consists of RGB LEDs (T‐1 3/4 (5 mm) full colour LED lamp, Kingbright, L‐154A4SURKQBDZGW) illuminating at 645, 515 and 460 nm respectively. The remaining three groups consist of T‐1 3/4, 5‐mm UV LEDs operating at wavelengths of 365, 390 and 395 nm. Both the LEDs and the camera are controlled by a Raspberry Pi 4B, enabling precise synchronisation and automated operation.

For each test, a fresh pollen load is placed at the centre of the box on a white paper disc, and image acquisition is triggered via the Raspberry Pi by a custom Python code. First, the red LEDs are lit for 0.13 s while the camera images the pollen load in 0.03 s. Then the system is idle for 0.2 s. The process is repeated sequentially with the other wavelengths (green, blue, UV 365, 390 and 395 nm). At the end of the sequence, we obtain six images corresponding to the six wavelengths of light. For each set of six images corresponding to a given pollen load, the red channel of the image captured under red LED illumination is segmented to identify the position and contour of the load, thus defining the region of interest (ROI) using an optimal threshold determined by OpenCV's thresholding functions in Python (https://opencv.org/). Subsequently, for each excitation wavelength, the RGB channel intensities are averaged within the ROI. Therefore, for each load, 18 measurement points are collected. These measurement points are encoded in the excitation wavelength‐pixel colour format. Pixel colours are abbreviated using the initial letter of each channel: green as ‘g’, blue as ‘b’ and red as ‘r’. For example, the intensity measurement in the red pixel after an excitation wavelength of 365 nm is denoted as 365r.

For each species, the 18 measurements from all analysed pollen loads are averaged and displayed on a radar chart with a logarithmic scale, generated using Excel software.

### Principal component analysis (PCA)

To further assess the discriminatory power of spectral data on pollen loads, dimensionality reduction was performed using PCA. The analysis was conducted on either the entire dataset or a subset of samples comprising species from the same family or pollen loads with similar colours. In each case, the raw spectra collected at six wavelengths were organised into a single data matrix, where each row corresponded to a single pollen load and each column represented fluorescence intensity for a given excitation and emission wavelength. PCA was implemented in Python using the scikit‐learn package (https://scikit‐learn.org/). Before analysis, the data were normalised, and a PCA model retaining 10 principal components was constructed. Across all PCA models presented, the first four principal components consistently explained at least 85% of the total variance. The score plots for PC1 vs PC2 are displayed to visualise the separation of pollen loads.

### Supervised classification analysis

Raw signal data acquired from both fluorescence spectroscopy and the pollen analyser were used as input for supervised classification algorithms. All classification experiments were conducted using the scikit‐learn library (https://scikit‐learn.org/). Before analysis, the data were normalised using the StandardScaler function. To identify the most effective classifier, we tested the following algorithms: Logistic Regression (LR), K‐Nearest Neighbors, Naive Bayes, Random Forest and Support Vector Machine (SVM) (Cortes & Vapnik, 1995). For each approach, hyperparameter optimisation was performed using the GridSearchCV function. Given the class imbalance in the datasets, a stratified 10‐fold cross‐validation was applied at each step of the hyperparameter search to ensure that each fold preserved the class distribution. In a multi‐class context, accuracy alone is insufficient for evaluating model performance. Therefore, we computed the following metrics for each class: (1) Recall = TP/(TP + FN), which represents the proportion of true positives (TP) relative to the total actual positives, that is, the sum of true positives and false negatives (FN). (2) Precision = TP/(TP + FP), which represents the proportion of true positives relative to the total predicted positives, that is, the sum of true positives and false positives (FP). (3) F1‐score, defined as the weighted harmonic mean of precision and recall, calculated as: F1 = 2 × (Precision × Recall)/(Precision + Recall). It ranges between 0 and 1, where higher values indicate a better balance between precision and recall. (4) Receiver Operating Characteristic – Area Under the Curve – One vs Rest (ROC‐AUC‐OVR) evaluates a model's ability to correctly distinguish between multiple classes. It measures how well the model ranks positive instances higher than negative ones across all class comparisons, using a ‘one‐vs‐rest’ (OVR) approach. A higher ROC_AUC_OVR score indicates better class separation, meaning the model assigns higher probabilities to the correct classes with minimal overlap. This metric is critical in our multiclass classification problem, especially given the slight overlap between classes observed in the PCA analysis.

To further understand the contribution of individual features and interpret model decisions in our best classifiers, we leveraged the SHapley Additive exPlanations (SHAP) method (Lundberg & Lee, 2017). This approach quantifies the average marginal contribution of each feature across all possible feature combinations, providing insight into feature importance. Two complementary metrics were employed: the mean absolute SHAP values, which quantify the overall influence of each feature on model predictions, regardless of direction and the mean SHAP values, which capture the average contribution of each feature, taking into account the direction of the effect (positive or negative impact). The 20 most influential features were identified based on their mean SHAP values, highlighting those with the most consistent impact on predictions. Each feature is named by combining the excitation and emission wavelengths in the format excitation_emission. The six excitation wavelengths (365, 390, 395, 460, 530 and 625 nm) are labelled L1–L6, respectively. For example, ‘L1_746’ refers to the fluorescence intensity at 746 nm emission under 365 nm excitation.

## Results

Raw data for pollen analyser and pollen spectrophotometry are given in Supporting Information Dataset S1.

### Cross validation of species identity

**Fig. 1:**
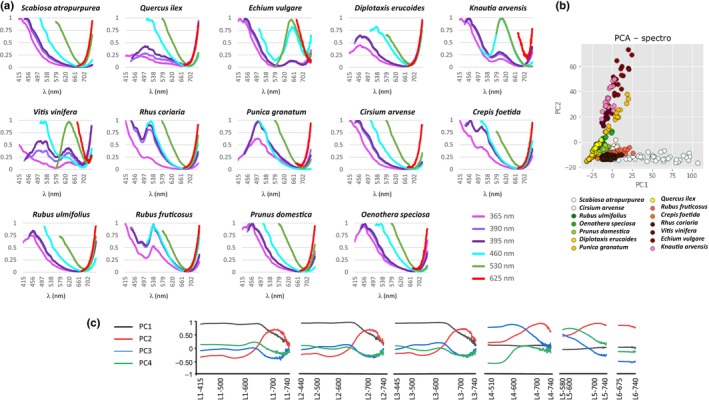
Spectral signature and principal component analysis (PCA). (a) Spectral signature of the 14 studied species. For each species, the six average emission spectra of the pollen load corresponding to the six excitation wavelengths have been combined. A visual comparison of these signatures highlights the specificity for each species. The vertical axis is normalised fluorescence intensity. (b) Score plot of PC1 vs PC2 for the PCA model built using 331 pollen loads from 14 different species. Each dot represents a pollen load colour‐coded according to its species, as indicated in the figure legend. While all species form distinct clusters, some exhibit partial overlap. (c) Loadings of the first four principal components (PC1–PC4) derived from the PCA of fluorescence spectral data. Each feature is named by combining the excitation and emission wavelengths in the format excitation_emission (Ln_λX). The six excitation wavelengths (365, 390, 395, 460, 530 and 625 nm) are labelled L1–L6, respectively. The curves represent the contributions of the first four principal components. For clarity, the feature plot is not organised by decreasing order of importance but rather grouped into subplots according to the corresponding excitation wavelengths (L1–L6). Positive or negative loadings indicate the direction and strength of each variable's contribution to the associated principal component. The distinct patterns across excitation_emission pairs reflect how spectral variables are weighted in the multivariate structure of the dataset.

From 7 to 15 June 2021, an average of 20 pollen loads of 14 species were collected manually from honeybees foraging on flowers of known species on several consecutive days, ensuring unambiguous species identification (see Fig. 1). Species identity was confirmed either through morphological analysis of the pollen grain using light microscopy or via the PlantNet application. The 14 species collected, which yield a set of pollen loads that are globally heterogeneous in size and colour, span a wide phylogenetic diversity, though seven of them belong to three families (three Rosaceae, two Asteraceae and two Caprifoliaceae).

### Pollen load analysis by fluorescence spectroscopy

Pollen, like most plant organs, is auto‐fluorescent upon excitation in a wide range of UV, visible and infrared wavelengths. Several studies have demonstrated that spectral signatures enable pollen identification, but current methods rely on fluorescence spectroscopy of soluble extracts, which are labour‐intensive and destructive, especially when the pollen source is honeybee‐collected pollen load. By contrast, we hypothesized that pollen spectral signatures could be produced directly from pollen load manually collected from the bees' legs, aiming for a non‐destructive approach. On the day of collection, each pollen load was analysed by fluorescence spectroscopy using six excitation wavelengths: 365, 390, 395, 460, 530 and 625 nm. For each species, all spectral data were normalised and averaged (Figs S1–S6).

For each species, the shape of the spectra – that is the number and positions of peaks – is reproducible, regardless of the excitation wavelength. However, for some species such as *D. erucoides*, the relative fluorescence intensities vary significantly from one load to another (Fig. S7). The reasons for such variability remain unclear. We verified that the variability in fluorescence intensity was not caused by the orientation of the pollen load in the well, the position of the well on the plate or the delay between collection and fluorescence measurement. Similarly, no correlation between pollen load size and fluorescence intensity was found (data not shown). The possibility of pollen bleaching due to light exposure can be excluded, as the pollen loads were kept in the dark immediately after collection. Despite this variability, we observed that each species presents a complex fluorescence spectrum with at least one main peak of fluorescence intensity. For example, the spectrum of *P. granatum* after excitation at 390 nm shows a single peak near 500 nm, but associated with one or two secondary peaks of fluorescence intensity. The spectrum of *K. arvensis* after excitation at 365 nm shows a maximum near 425 nm, a clear secondary peak near 445 nm and a final broader peak near 595 nm. The superposition of the average fluorescent spectra of each of the 14 pollen load species for each excitation wavelength is shown in Fig. S8. These panels illustrate that the 14 species have specific emission profiles with distinctive spectral features, including different fluorescence peaks or different ratios of these peaks. For each species, the combination of data from these six spectra forms a spectral signature (Fig. 1a). Spectral signature differences can be visually clear in some cases but subtle in others, requiring a quantitative assessment of spectral specificity.

### Principal component analysis

Given the complexity and large number of parameters in our dataset, clustering the data using PCA reduces this dimensionality by linearly transforming the data while capturing the most significant variations, making it easier to visualise clustering patterns and compare species data. The raw spectra of each pollen load, collected from the six excitation wavelengths, were concatenated into a single data matrix. The PCA model was elaborated with 10 principal components (PC), four of which explain 90.1% of the variance and whose loadings are presented in Fig. 1(c). These loading profiles reveal distinct patterns across excitation‐emission pairs, meaning different spectral regions contribute in complementary ways to species‐level discrimination. Plotting PC1 vs PC2 led to the separation of the pollen load spectral data into clusters corresponding to each pollen species (Fig. 1b). This supports that the six fluorescence spectra constitute a spectral signature for each pollen species, although on the 2D graph some of the clusters partially overlap.

As one limitation of pollen identification by chromatic assessment is to distinguish pollen loads of similar colours, it is interesting to evaluate how our method is able to discriminate such pollen loads. To determine the potential link between the spectral pattern of pollen loads and their colour, we first visually classified our collection of pollen loads according to tone. We obtained five groups: two species with white or very light pollen loads (*S. atropurpurea* and *C. arvense*), three species with yellow pollen loads (*Q. ilex*, *D. erucoides* and *P. granatum*), two species with orange pollen loads (*R. fruticosus* and *C. foetida*), three species with green pollen loads (*R. ulmifolius*, *P. domestica* and *O. speciosa*) and three species with black or very dark pollen loads (*E. vulgare*, *V. vinifera* and *R. coriaria*). We then superimposed the averaged spectra of the species in each of these tone categories (Fig. S9). Although spectra from the same taxonomic groups exhibit clear divergences that could potentially enable differentiation of pollen loads by species, we generally observe similar spectral patterns for pollen loads of the same colour. This similarity is particularly evident between *P. domestica* and *O. speciosa*, and even more pronounced between *S. atropurpurea* and *C. arvense*. However, the PCA analysis reveals that within each tone group, species have been distinctly clustered, and even a two principal components representation provides a fairly sufficient degree of separation to accurately identify each species (Fig. S9).

**Fig. 2:**
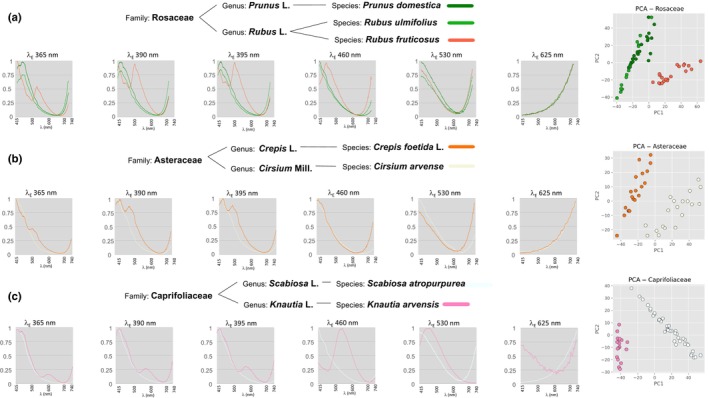
The discriminative power of the method of pollen load analysis by fluorescence spectroscopy allows identification at the species level. A visual comparison of the fluorescence spectra for species within the same family is presented for each excitation wavelength. Principal component analysis (PCA) applied to each family permits the quantification and the illustration of the variation between species. Three families are analysed: Rosaceae (a), Asteraceae (b) and Caprifoliaceae (c). Within each family, species can be distinguished and classified either through direct visual comparison of their spectra or PCA analysis.

Similarly, the classification of the spectra according to their phylogenetic distances illustrates the resolving taxonomic power of the method. Among the 14 species analysed, the families Asteraceae and Caprifoliaceae are represented by two species, and the family Rosaceae has three members, two of which are of the same genus, *Rubus* (other families were represented by a single species). The spectra of each of these species are superimposed by family (Fig. 2). In the case of the Asteraceae and Caprifoliaceae, the two species exhibit distinct peaks, allowing pollen load classification by spectral comparison, as confirmed by well‐separated PCA clusters in 2D (Fig. 2). In the Rosaceae family, *P. domestica* and *R. ulmifolius* have the most similar spectra; yet PCA analysis provides sufficient distinction to reliably differentiate each species, as shown in the two‐component representation. Moreover, the two *Rubus* species display distinct spectral peaks, suggesting the method is able to achieve species‐level taxonomic resolution (Fig. 2).

### Supervised classification analysis

**Fig. 3:**
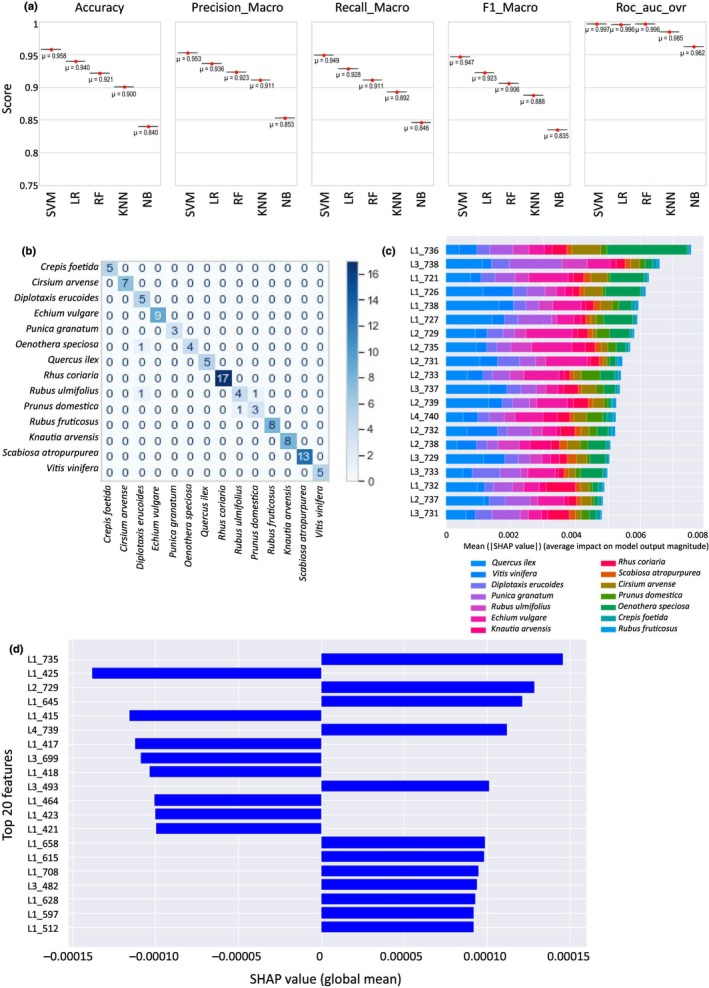
Supervised classification analysis. (a) Main metrics for comparing the five tested classifiers. Support Vector Machine (SVM) achieves the best performance across all metrics. (b) Confusion matrix for the SVM classifier illustrating the model performance. The true labels are listed on the vertical axis, while the predicted labels are on the horizontal axis. Each cell represents the number of instances for the corresponding true–predicted label pair, with diagonal elements indicating correct classifications. The colour intensity bar indicates the scale of the counts. (c) Bar plot of mean absolute SHAP values across different classes. Each bar represents the average impact of a feature on the model's output magnitude for a specific class, with colours indicating the respective classes. The *x*‐axis denotes the mean absolute SHAP value, indicating the relative importance of each feature in the classification task. (d) Global SHAP summary plot of the top 20 wavelengths with the greatest impact on the classification model. This plot highlights the most influential wavelengths, showing their contribution to the model's predictions.

**Fig. 4:**
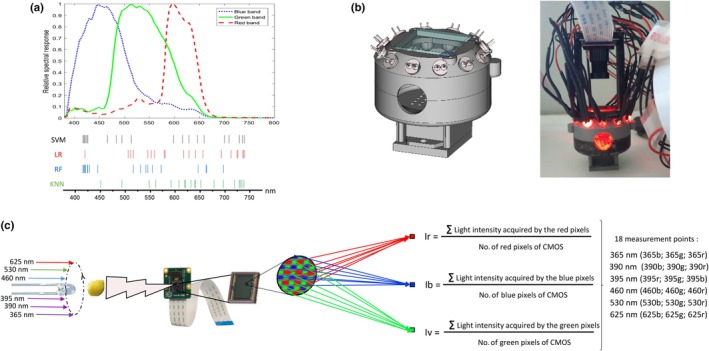
The pollen analyser prototype. (a) Combination of the spectral response curves of the sensor Sony IMX219 used in Raspberry Pi v.2 NoIR camera (derivate from Pagnutti *et al*., 2017) and the 20 most significant wavelengths, represented as vertical lines, identified as the most relevant features by each supervised classification model. The horizontal axis indicates the emission wavelength (in nm). Note that the spectral response is made with the Raspberry Pi v.2 camera which includes an IR filter. However, our prototype uses the NoIR model which is a Sony IMX219 sensor without such an IR filter on the lens, extending the vision from visible to the near‐infrared. As a result, radiation beyond 650 nm can still be recorded with a satisfactory sensitivity. (b) 3D representation of our pollen analyser prototype (left) alongside a photograph of the final version now equipped with the camera and necessary connections. (c) A schematic representation of the data acquisition method detailing the nomenclature of the 18 measurement points of our pollen analyser.

Having established by fluorescence spectroscopy and PCA analysis that pollen loads exhibit species‐specific spectral signatures, we then developed an automated method for classifying pollen loads and reliably predicting their species identity. Since our dataset is already labelled with species identities, we trained several supervised learning models on a dataset derived from 70% of the spectral data obtained from pollen load collection. All classifiers, except Naive Bayes, achieve strong performances with accuracy exceeding 90%. These high levels of accuracy underscore the robustness of spectral signatures as reliable taxonomic markers, highlighting their potential for precise species identification. Among all models, the SVM classifier outperformed the others, achieving the highest classification accuracy – over 95% after hyperparameter optimisation – and the highest ROC_AUC_OVR score of 0.997, demonstrating its ability to effectively separate classes (Fig. 3a). The final optimised model used a linear kernel with *C* = 0.1 and gamma = ‘scale’. The resulting confusion matrix is presented Fig. 3(b). The vertical axis represents the true species identities, while the horizontal axis corresponds to the predicted species identities. The poorest classification performance was observed for *D. erucoides*, which was misclassified as *O. speciosa* and *R. ulmifolius*. Similarly, *Prunus fructicosus* and *R. ulmifolius*, belonging to the same genus, remain difficult to distinguish, as expected from their slightly overlapping clusters observed in the PCA analysis (Fig. 2a). However, their classification accuracy remains above 0.75. To further interpret the model's predictions, we employed the SHapley Additive Explanations (SHAP) method (Lundberg & Lee, 2017) to quantify and rank the wavelengths based on their influence on the model's predictions. A global analysis of the SHAP value distribution (Fig. 3c) indicates that no single feature exhibits a dominant colour, meaning that multiple features contribute broadly across different classes. However, some features still have a stronger positive contribution for specific classes – for example, L1_736, which contributes more to the classification of *R. coriaria* than to other classes. The highest SHAP values correspond to the excitation_emission wavelength pairs that have the greatest impact on the predictive model. However, these SHAP values remain small, at the 10^−3^ level, indicating that their overall impact on predictions is subtle. These observations suggest that our model, which relies on a large combination of features to predict species identity, is complex and robust enough to generalise well when incorporating new data. Sorting the 20 features with the highest global impact on the SVM predictive model (Figs 3d, 4a) reveals that the decisive wavelengths of the UV/visible emission spectra are clustered into three main regions: *c*. 450 nm (415–512 nm), 625 nm (597–658 nm) and 720 nm (699–739 nm). Comparing these 20 highest SHAP values across other classifiers with over 90% accuracy confirms this clustering pattern but with slightly different wavelength intervals, suggesting that other wavelengths across the entire UV/visible spectrum also play a significant role in species classification (Fig. 4a).

### Development of a pollen analyser prototype

Based on the previous results, we have therefore developed a new methodology able to analyse and classify pollen loads using a system that is more affordable than a fluorescence spectrometer, easy to use and portable. The general principle of our pollen analyser is to collect the radiation emitted by a pollen load using a camera during a short pulse of exciting light produced by various monochromatic LEDs. Conceptually, our pollen analyser records a subsample of the fluorescence spectrum of a pollen load, but with a resolution of 75–100 nm corresponding to the bandwidth of each camera filter. As one of our primary goals is to create a low‐cost system, we chose to leverage the Raspberry technology, combining a Pi computer with a v.2.1 NoIR camera that provides raw image data. The Raspberry v.2.1 NoIR camera uses a Sony IMX219 sensor whose characteristics meet our needs, in particular the spectral sensitivity characteristics covering the three spectral areas identified with SHAP values (Pagnutti *et al*., 2017; Fig. 4a). However, SHAP values analysis of SVM and LR models highlight the importance of emission wavelengths in the deep red. Consequently, the camera's infrared filter has been removed to allow the capture of infrared radiation. These SHAP values further demonstrate that, among all classifiers achieving accuracy rates over 90%, all the excitation wavelengths employed are critical; even the 465 nm (L1) is the most critical for species classification (data not shown). Consequently, the same six excitation wavelengths are retained for the prototype. The prototype of this analysis tool is 3D printed (Fig. 4b) and controlled via the Raspberry Pi 4, which turns the LED on and off and manages image acquisition for data collection. In practice, a pollen load deposited in the round chamber of our analyser is illuminated sequentially, for less than a second, by quasi‐monochromatic light of 365, 390, 395, 460, 530 and 625 nm. Each time, an image is recorded. Each of the six images corresponding to the different wavelengths is segmented and analysed by calculating the average light intensity obtained for each of the three types of pixels within the load ROI: blue, green and red. In this way, each pollen load is characterised by 18 measurement values (Fig. 4c).

**Fig. 5:**
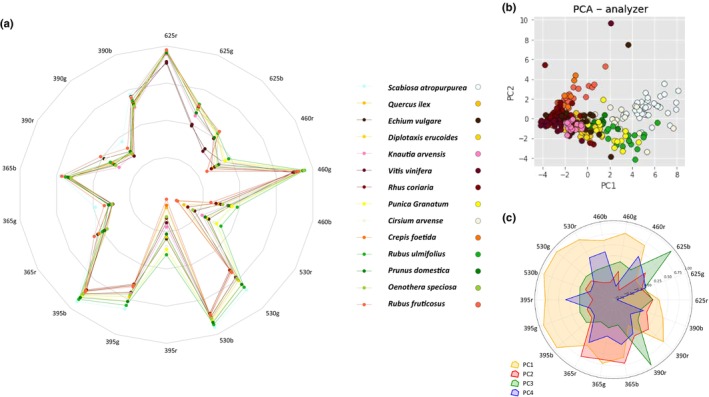
Results from the pollen analyser. (a) Superposition of the average radar graphs for the 14 species studied. A careful observation allows one to notice that each radar graph constitutes a specific signature of the species. (b) Score plot of principal component (PC1) vs PC2 for the principal component analysis (PCA) model built using 334 pollen loads from 14 different species analysed with the pollen analyser. Each dot represents a pollen load that is labelled by its species colour. Species colours are indicated in the figure legend. While all species are generally clustered, the clustering is less efficient for some species and the overlap between clusters is more pronounced than with the fluorescent spectroscopy data. (c) Radar plot comparing the loadings of the first four principal components (PC1–PC4), which together explain > 85% of the total variance across 18 spectral variables. Each axis represents a variable (e.g. 625r and 530v) and the length and direction of the loadings indicate the relative contribution of each variable to each component.

The same set of pollen loads from 14 species used for fluorescence spectroscopy analysis was also recorded on the day of collection with the pollen analyser. For each species, the average of all measurement points is calculated and the data are represented with a radar chart using a logarithmic scale (Fig. S10). Interestingly, as for the fluorescence spectroscopy approach, several species present variability for several measurement points. The reasons for these discrepancies are not clear yet. Still, we observe that the tendency to get more variable data is more pronounced when the pollen load colours darken. This phenomenon is maximal with *E. vulgare* whose pollen loads are the blackest of our samples (Fig. S11). The superposition of the 14 species illustrates that each radar chart forms a signature of the foraged plant (Fig. 5a).

**Fig. 6:**
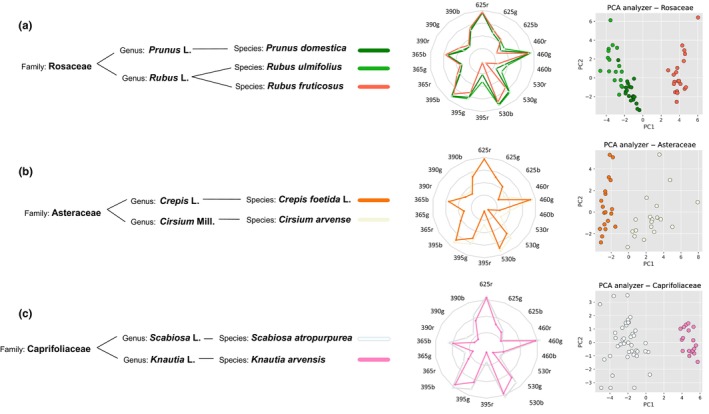
The discriminatory power for taxonomic classification of pollen loads of our pollen analyser approach reached the species rank. The visual comparison of radar charts of species from the same family is presented. Principal component analysis (PCA) for each family enables the quantification and the illustration of the variation between species. Three families are analysed: Rosaceae (a), Asteraceae (b) and Caprifoliaceae (c). In each family, the samples could be discriminated and classified by visual comparison of the spectra or PCA analysis.

Considering the substantial reduction in the number of recorded values compared to spectroscopic data (1381 features for fluorescence spectroscopy vs 18 for the pollen analyser), it was expected that the variations in signature shape between species were less pronounced. Nevertheless, the corresponding PCA model, which explains > 85% of the variance with four principal components whose main loadings are presented in Fig. 5(c), demonstrates that the data extracted from the pollen analyser are organised into clusters as shown by the score plots of PC1 vs PC2 (Fig. 5b). However, some clusters, such as that of *P. granatum*, are more diffuse compared to the fluorescence spectroscopy data, and the overlap between clusters is slightly more pronounced. Likewise, the radar charts for species with similar pollen load hues reveal consistent patterns, suggesting that colour information is embedded in the shape of the pollen signature (Fig. S12). Consequently, the PCA analysis of these species shows relatively broader clustering with frequent overlap, indicating that the pollen analyser is less effective at distinguishing pollen loads with similar colours (Fig. S12). By contrast, the superimposition of radar charts for species within the same family highlights the lack of correlation between related species and the shape of the radar chart (Fig. 6). For instance, *R. fruticosus* and *R. ulmifolius* within the genus *Rubus* exhibit distinct patterns, demonstrating the resolving power of our pollen analyser at the species level, which is consistent with results previously obtained using fluorescence spectroscopy analysis.

**Fig. 7:**
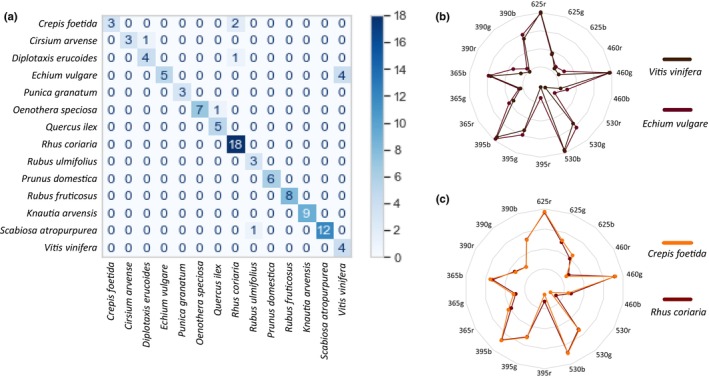
Classification from pollen analyser data. (a) Confusion matrix for the prediction of the pollen load botanical source using our Support Vector Machine model applied to a given set of the pollen analyser data for the 14 species studied. Rows represent the real species and columns represent the predicted species. The intensity bar represents the scale. The recognition rate is 91%. The worst prediction is obtained for *Vitis vinifera*, *Echium vulgare* and *Crepis foetida*. (b) Superposition of the *V. vinifera* and *E. vulgare* radar charts. (c) Superposition of the *C. foetida* and *Rhus coriaria* radar charts.

To enable the automatic identification of the plant source of pollen loads using the pollen analyser, we applied the same machine learning strategies as those used with fluorescence spectroscopy data. Once again, the SVM classifier outperformed the other models. While the ROC_AUC_OVR scores for Random Forest, SVM and LR are all closely aligned *c*. 0.99, SVM is the only model to exceed 90% accuracy, achieving 91.0% on the test data following hyperparameter optimisation (data not shown). This result closely approximates the accuracy obtained from the model trained on fluorescence spectroscopy data. Analysis of the corresponding confusion matrix reveals that the lowest precision occurs for *V. vinifera*, due to frequent misclassification of *E. vulgare* as *Vitis* (Fig. 7a). Although their radar charts display subtle differences (Fig. 7b), the discrimination between these species, characterised by darker pollen colours, is more challenging due to the high variability in the collected data. Another frequently misclassified species is *C. foetida*, often confused with *R. coriaria* and whose spectral signatures are highly similar (Fig. 7c).

## Discussion

This study demonstrates that pollen grains analysis for taxonomic classification can be extended to pollen loads. By using UV/VIS fluorescence spectroscopy, we were able to perform the first systematic characterisation of the autofluorescent properties of the pollen load of 14 different species. The fluorescence spectra of the pollen loads, measured at six different excitation wavelengths, from 365 to 625 nm, provide a distinct signature for each species. Moreover, we were able to exploit these interspecific spectral differences to classify automatically pollen load according to their species using a supervised classification methods.

The spectroscopic properties of the pollen load are derived from the chemical composition of the pollen grains. While > 200 compounds have been found in bee pollen, the chemical composition of the pollen load varies significantly depending on its botanical origin (Spulber *et al*., 2018; Prdun *et al*., 2021). Specifically, the presence of bioactive compounds, such as phenolics, flavonoids and alkaloids, or those involved in pollen odour, or their ratios, is highly variable between species (Modro *et al*., 2009; Gabriele *et al*., 2015; Thakur & Nanda, 2020). These compounds have already been used to determine the floral source of pollen grain using several biochemical methodologies (Dobson *et al*., 1996; Campos *et al*., 1997). As many of these compounds are fluorophores, our approach also capitalises on these species‐specific chemical characteristics, which explains the intra‐ and inter‐specific variability we observed. Indeed, spectral analysis of pollen grains has already shown that the main spectral properties originate from the specific ratio between these fluorophores for each species. The most prominent peaks are located near 415–425 nm, 440–460 nm and above 740 nm. These peaks may correspond to, respectively, terpenoids, flavonoids and Chl (Buschmann *et al*., 2001; García‐Plazaola *et al*., 2015; Talamond *et al*., 2015). However, it remains speculative to directly correlate the fluorescence spectral profiles to chemical composition or to attribute fluorescent peaks to corresponding fluorophores.

Regarding species identification, the choice of wavelengths is critical. Dunker *et al*. (2021) found that merging brightfield and fluorescence information is the most efficient way to classify pollen grain, with the best combination of fluorescence being excitation at 488 nm and emission *c*. 528 nm. By contrast, emission in the red spectral region after excitation by 488 nm is the least distinctive combination (Dunker *et al*., 2021). In this analysis, we determined that the least discriminating combination was 625 nm excitation/675–740 nm emission, as the species were separated into two groups: one group of species with a peak below 675 nm and another with a peak above 740 nm. The SHAP analysis from the different models indicated that the most discriminating wavelengths come from spectral data obtained with excitation at 365 nm. This observation is further supported by the decrease in accuracy values for supervised classification models trained on data from a single excitation wavelength, with performance declining as the excitation wavelength increases (data not shown). Interestingly, if, as in our experiment, the number of plant species studied is limited, working with a single excitation wavelength of 365 nm may be sufficient for plant species identification. Therefore, plant species identification from pollen loads may require simpler instrumentation than pollen grain characterisation. Our pollen analyser prototype, designed for low‐cost, lightweight and field‐deployable taxonomic assignment, represents the simplest possible attempt at achieving this goal. It emits radiation using a subset of six monochromatic stimuli previously used in fluorescence spectroscopy analysis. In fact, we have established that a combination of a rudimentary computer vision system and a supervised classification can effectively classify pollen loads. Thus, the pollen analyser is a reliable and promising device to follow plant flowering directly from foraged pollen. Our prototype provides a proof of concept for species sorting but requires a large database of analysed pollen loads to be operational for ecological studies. However, it is suitable for many field conditions and can be widely distributed to scientific or professional communities or for citizen science experiments. Such deployment would support the creation of a large database of pollen loads, enhancing species identification and enabling time‐ and space‐resolved flowering tracking.

A central question concerns the evaluation of the taxonomic resolution of our approaches. Species‐level identification has rarely been achieved using automated image analysis systems (Dunker *et al*., 2021). With a limited number of 14 species, we were able to discriminate between species with the same genus, *Rubus*. Moreover, spectroscopy allowed us to distinguish two closely related species of the caprifoliaceae family, *S. atropurpurea* and *K. arvensis*, which have very similar flowers and whose pollen grains are indistinguishable from each other with the classical light microscopy approach. Future work should include more congeneric species to clarify the potential and limitations of our approaches. Nevertheless, although a larger sample size is needed for definitive conclusions, it appears that our system has similar resolving power as existing methods, but, unlike others, it offers the advantage of being non‐invasive. Keeping the pollen load as an intact botanical object is decisive for the construction of databases but also for reliable comparative studies between laboratories and ecosystems. This approach will allow ecological and physiological studies on the adaptation of pollen traits (Davis, 2009).

One challenge of this study is the intraspecific variability observed in the spectroscopic and pollen analyser data. These variations as well as their origins are difficult to interpret since they are only observed for certain species. Pöhlker *et al*. (2013) demonstrate that the autofluorescence intensity of pollen grains can vary between freshly collected samples and those stored for a longer period, within the same species (Pöhlker *et al*., 2013; Walther *et al*., 2025). Such factors could also have influenced measurement, at the macroscopic level, as a pollen load consists of a large collection of pollen grains likely collected from different flowers, and potentially at different developmental ages. However, the magnitude of variation in pollen loads is expected to be lower than that in individual pollen grains, as the autofluorescence properties of individual grains are averaged at the pollen load level. Indeed, we observe that the fluorescence intensities of the pollen loads fluctuate within a range of 5–10 d for most of the species studied (data not shown). To minimise potential bias in species identification, all data used in this study were recorded < 4 h after pollen load collection. This permits excluding this potential bias in species identification. Importantly, the variability in fluorescence intensity was not caused by the orientation of the pollen load in the well, the position of the well on the plate or the age of the pollen load after harvest. Similarly, no correlation between pollen load size and fluorescence intensity was found (data not shown).

Another challenge is the presence of polyfloral loads. While the vast majority of pollen loads were monospecific, some were identified as polyfloral. The spectral characteristics of these polyfloral loads likely reflect the relative proportions of each floral origin. If one species is dominant within a load, the presence of less abundant pollen grains could contribute to the observed intra‐specific variations. However, this hypothesis requires further investigation in future studies. In a hypothetical scenario where the pollen proportions are more balanced, the resulting autofluorescence properties could potentially be misinterpreted as belonging to a distinct species. From a plant community perspective, accurately identifying polyfloral loads could provide valuable insights into the spatial distribution of plant species in the field, such as revealing co‐flowering species or plants growing in a close proximity. However, reliable classification of polyfloral loads remains challenging and requires further research.

Despite these intra‐specific variations, automatic classification was achieved with high accuracy. Notably, an accuracy exceeding 90% is sufficient to monitor seasonal flowering peaks of different species and track how flowering patterns evolve over extended periods. Given that the foraging radius of bees ranges from *c*. 1.2 to 12 km (Visscher & Seeley, 1982; Beekman & Ratnieks, 2000; Steffan‐Dewenter & Kuhn, 2003), our approach has the potential to monitor plant communities across areas spanning 3.77–314 km^2^. Due to the simplicity and affordability of our device, its large‐scale deployment across diverse biomes and extensive territory is a realistic prospect. This could be facilitated through collaborations with beekeepers or integration into citizen science initiatives. The accessibility and scalability of this method represent a significant advantage outweighing the slight reduction in accuracy.

Furthermore, as pollen loads contain essential amino acids, saturated/unsaturated fatty acids, carbohydrates, minerals, vitamins and various compounds with antioxidant or anti‐inflammatory activities, they also constitute natural therapeutic alternatives against pathologies such as prostatic hyperplasia (Campos *et al*., 2010; Denisow & Denisow‐Pietrzyk, 2016). These nutritional and bioactive properties are species‐specific. Consequently, the provenance and authenticity of pollen load are key parameters for the pharmaceutical, nutraceutical, food and cosmetic industries that market or utilise bee pollen. Our non‐destructive approach offers a valuable tool for these industrial applications. Since such uses demand high classification accuracy, the algorithm's performance could be further optimised by expanding the size of the training dataset. Additionally, for applications focusing on specific pollen types, the algorithm could be fine‐tuned to enhance accuracy for the species of interest.

Our pollen analyser identifies the floral source of pollen loads collected by honeybees with slightly lower reliability than a spectrofluorometer. However, its success rate remains sufficient for confident use in ecological studies, while offering greater deployability in the field. A key future perspective is to automate our pollen analyser while keeping the system non‐invasive. This could be achieved by analysing pollen loads directly from the corbicula of forager bees returning to the hive, effectively making the bees themselves the agents positioning the pollen loads for analysis. Outfitting beehives with such a device could transform them into observatories of insect‐pollinated plant communities, providing valuable insights in the context of global change. Furthermore, automation would enable the continuous monitoring of foraging activity, allowing one to extrapolate flowering dynamics, generating quantitative data for ecological studies. Additionally, honeybee colonies have experienced increasing mortality in recent decades, with multiple factors contributing to their decline, including nutrient stresses (Woodcock *et al*., 2022). In this context, in addition to understanding the foraging preference at the colony level, our device could play a crucial role in real‐time monitoring of a colony's floral resource diversity and identifying periods of nutrient stress.

## Competing interests

None declared.

## Author contributions

MR, P‐OC and JB were involved in conceptualization and methodology. CL, AC, P Chevin, L‐PD and CC were involved in investigation. CMD, PP, DJ, J‐BT, P Charnet and MR were involved in data curation and formal analysis. MR was involved in writing – original draft. P‐OC; CMD; JB; DJ, P Charnet and J‐BT were involved in writing – review and editing. MR and P‐OC contributed equally to this work.

## Disclaimer

The New Phytologist Foundation remains neutral with regard to jurisdictional claims in maps and in any institutional affiliations.

## Supporting information


**Dataset S1** Data pollen analyser and data pollen spectrophotometry for the 14 studied species.

**Fig. S1** Normalised average fluorescence responses of the 14 species studied induced by an excitation at 365 nm acquired through fluorescence spectroscopy.
**Fig. S2** Normalised average fluorescence responses of the 14 species studied induced by an excitation at 390 nm acquired through fluorescence spectroscopy.
**Fig. S3** Normalised average fluorescence responses of the 14 species studied induced by an excitation at 395 nm acquired through fluorescence spectroscopy.
**Fig. S4** Normalised average fluorescence responses of the 14 species studied induced by an excitation at 460 nm acquired through fluorescence spectroscopy.
**Fig. S5** Normalised average fluorescence responses of the 14 species studied induced by an excitation at 530 nm acquired through fluorescence spectroscopy.
**Fig. S6** Normalised average fluorescence responses of the 14 species studied induced by an excitation at 625 nm acquired through fluorescence spectroscopy.
**Fig. S7** Normalised fluorescence spectra (in grey shade) acquired through fluorescence spectroscopy and average fluorescence spectra (in red) induced by an excitation at 365 nm for 6 species showing the variability obtained with some species.
**Fig. S8** Superposition of the average fluorescence spectra acquired through fluorescence spectroscopy of the 14 species studied induced by an excitation wavelength of 365, 390, 395, 460, 530 or 625 nm.
**Fig. S9** Superposition of the average spectra acquired through fluorescence spectroscopy for each excitatory wavelength of species with pollen load with the same tone.
**Fig. S10** Radar representation of the average data collected with the pollen analyser for each species.
**Fig. S11** Superposition of the radar graphs of data acquired with the pollen analyser for each pollen load for a given species (in grey) and the average radar graph for this species (in red).
**Fig. S12** Superposition of the radar graphs of data acquired with the pollen analyser for different species, grouped by similar pollen load colour tones.
Please note: Wiley is not responsible for the content or functionality of any Supporting Information supplied by the authors. Any queries (other than missing material) should be directed to the *New Phytologist* Central Office.

## Data Availability

All data supporting the findings are available as Supporting Information (Dataset S1).
