# Supplementary material for: Automated identification of honey bee pollen loads for field‐applied palynological studies
Source: New Phytol. 2025 Aug 2;248(3):1584–99. doi: 10.1111/nph.70435 (PMC12489280; doi:10.1111/nph.70435)

New Phytologist Supporting Information

Article title: Automated identification of honey bee pollen loads for field-applied palynological studies

Authors: Jonathan Barés, Pascal Poncelet, Christine M Doucet, Charline Legrand, Anais Cambon, Capucine Carlier, Patrick Chevin, Leo-Paul Dewaele, Delphine Jullien, Jean-Baptiste Thibaud, Pierre Charnet, Matthieu Rousset and Pierre-Olivier Cheptou

Article acceptance date: 15 July 2025

Supplementary figure 1: Normalized average fluorescence responses of the 14 species studied induced by an excitation at 365 nm acquired through fluorescence spectroscopy.

Supplementary figure 2: Normalized average fluorescence responses of the 14 species studied induced by an excitation at 390 nm acquired through fluorescence spectroscopy.

Supplementary figure 3: Normalized average fluorescence responses of the 14 species studied induced by an excitation at 395 nm acquired through fluorescence spectroscopy.

Supplementary figure 4: Normalized average fluorescence responses of the 14 species studied induced by an excitation at 460 nm acquired through fluorescence spectroscopy.

Supplementary figure 5: Normalized average fluorescence responses of the 14 species studied induced by an excitation at 530 nm acquired through fluorescence spectroscopy.

Supplementary figure 6: Normalized average fluorescence responses of the 14 species studied induced by an excitation at 625 nm acquired through fluorescence spectroscopy.

Supplementary figure 7: Normalized fluorescence spectra (in grey shade) acquired through fluorescence spectroscopy and average fluorescence spectra (in red) induced by an excitation at 365 nm for 6 species showing the variability obtained with some species. For example, the spectra obtained from *Scabiosa atropurpurea* pollen loads are highly reproductible while those of *Diplotaxis erucoides* are more variables.

Supplementary figure 8: Superposition of the average fluorescence spectra acquired through fluorescence spectroscopy of the 14 species studied induced by an excitation wavelength of 365nm, 390 nm, 395 nm, 460 nm, 530 nm or 625 nm. These representation illustrate that the species present specific peak of fluorescence.

Supplementary figure 9: Superposition of the average spectra acquired through fluorescence spectroscopy for each excitatory wavelengths of species with pollen load with the same tone. The PCA clustering of each color tone group is also presented. This approach provides an unbiased way to separate pollen loads of the same color tone.

Supplementary figure 10: Radar representation of the average data collected with the pollen analyzer for each species.

Supplementary figure 11: Superposition of the radar graphs of data acquired with the pollen analyzer for each pollen load for a given species (in grey) and the average radar graph for this species (in red). Species present a limited data variability like *Prunus domestica* or *Scabiosa atropurpurea* but for others species the data variability is larger like *Echium vulgare* or *Rhus coriara*.

Supplementary figure 12: Superposition of the radar graphs of data acquired with the pollen analyzer for different species, grouped by similar pollen load color tones. The PCA clustering of each color tone group is also presented.

SUPPLEMENTARY FIGURE 1 (Excitation wavelength 365nm)

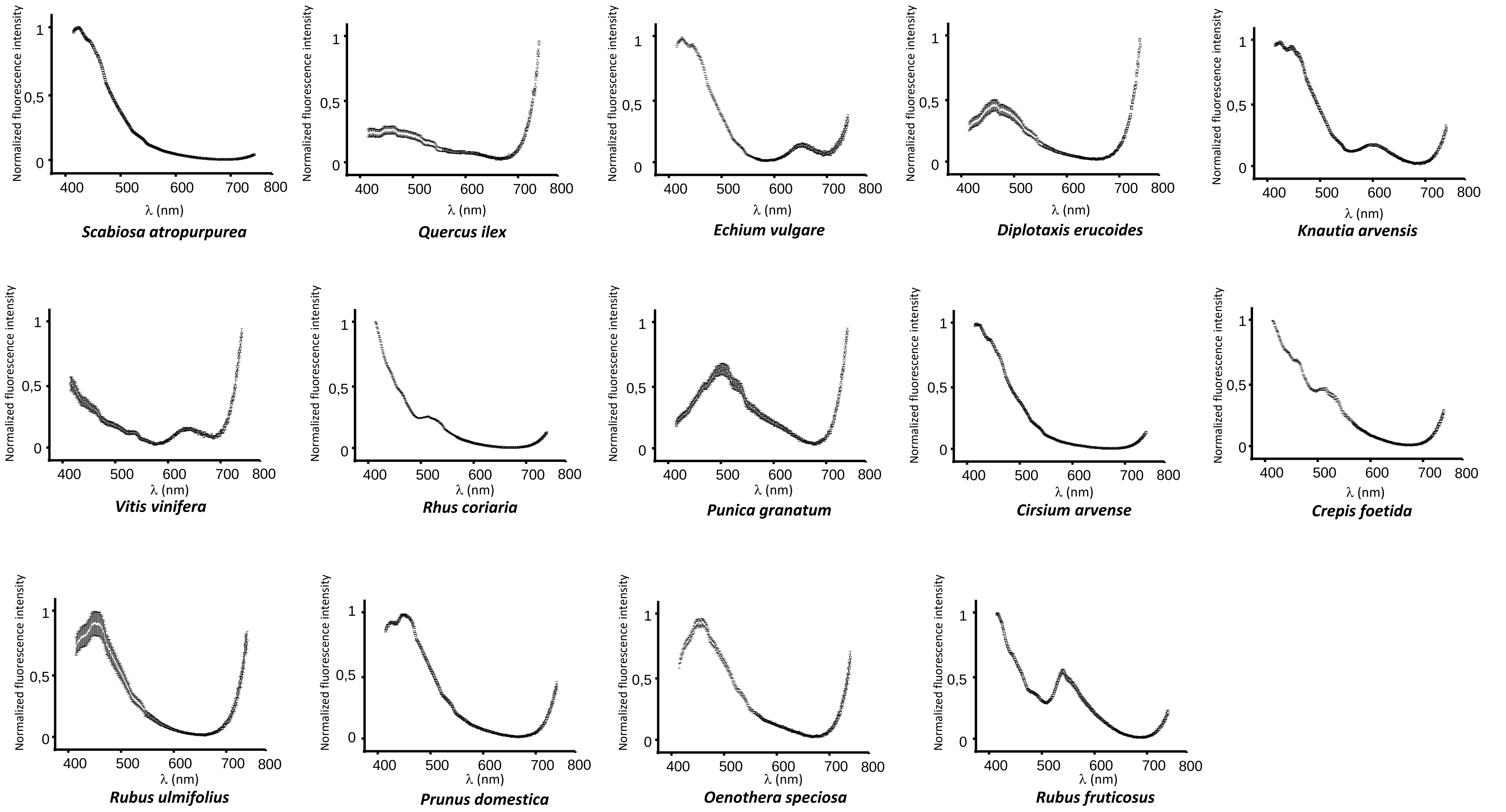

SUPPLEMENTARY FIGURE 2 (Excitation wavelength 390nm)

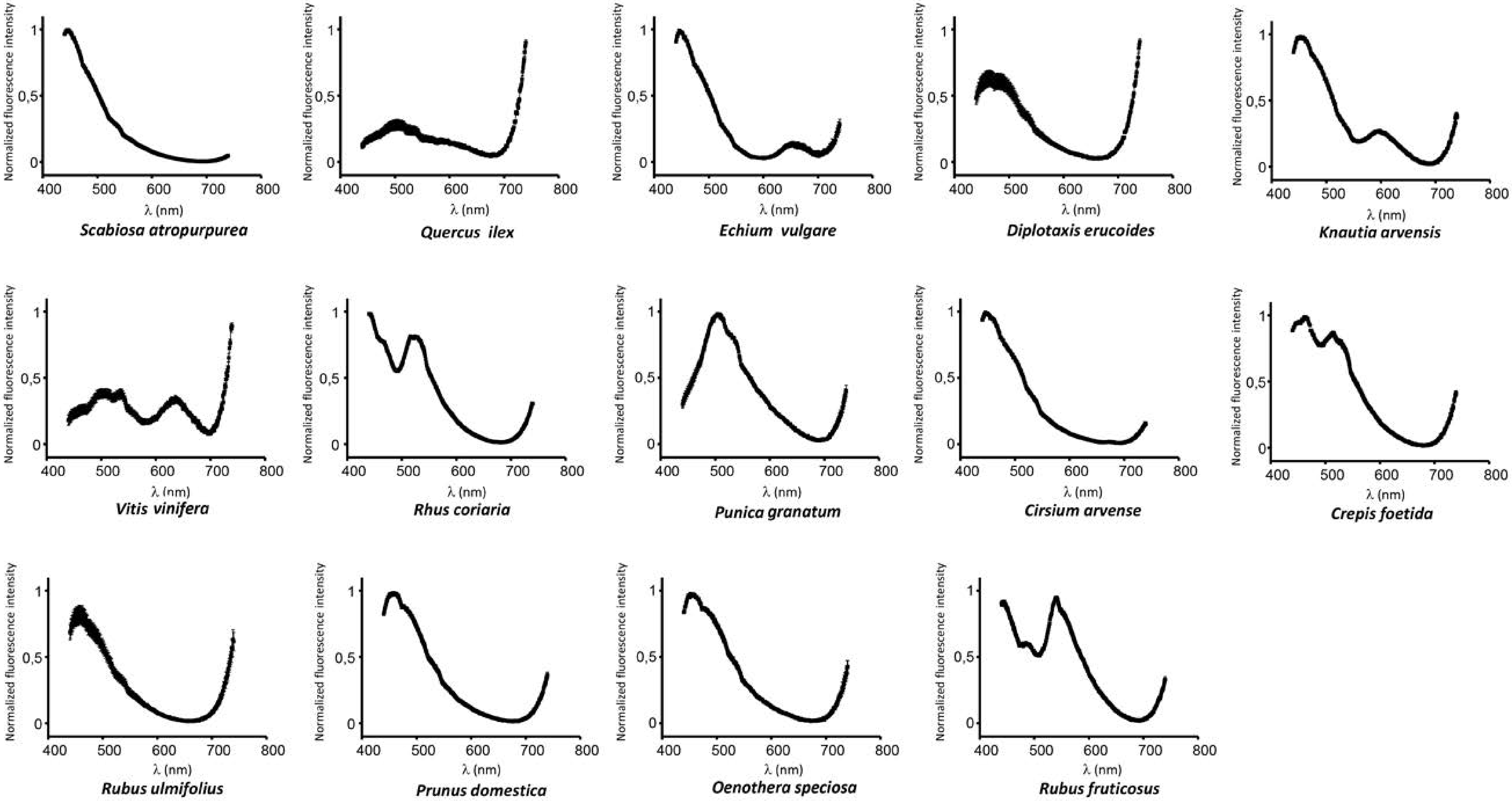

SUPPLEMENTARY FIGURE 3 (Excitation wavelength 395nm)

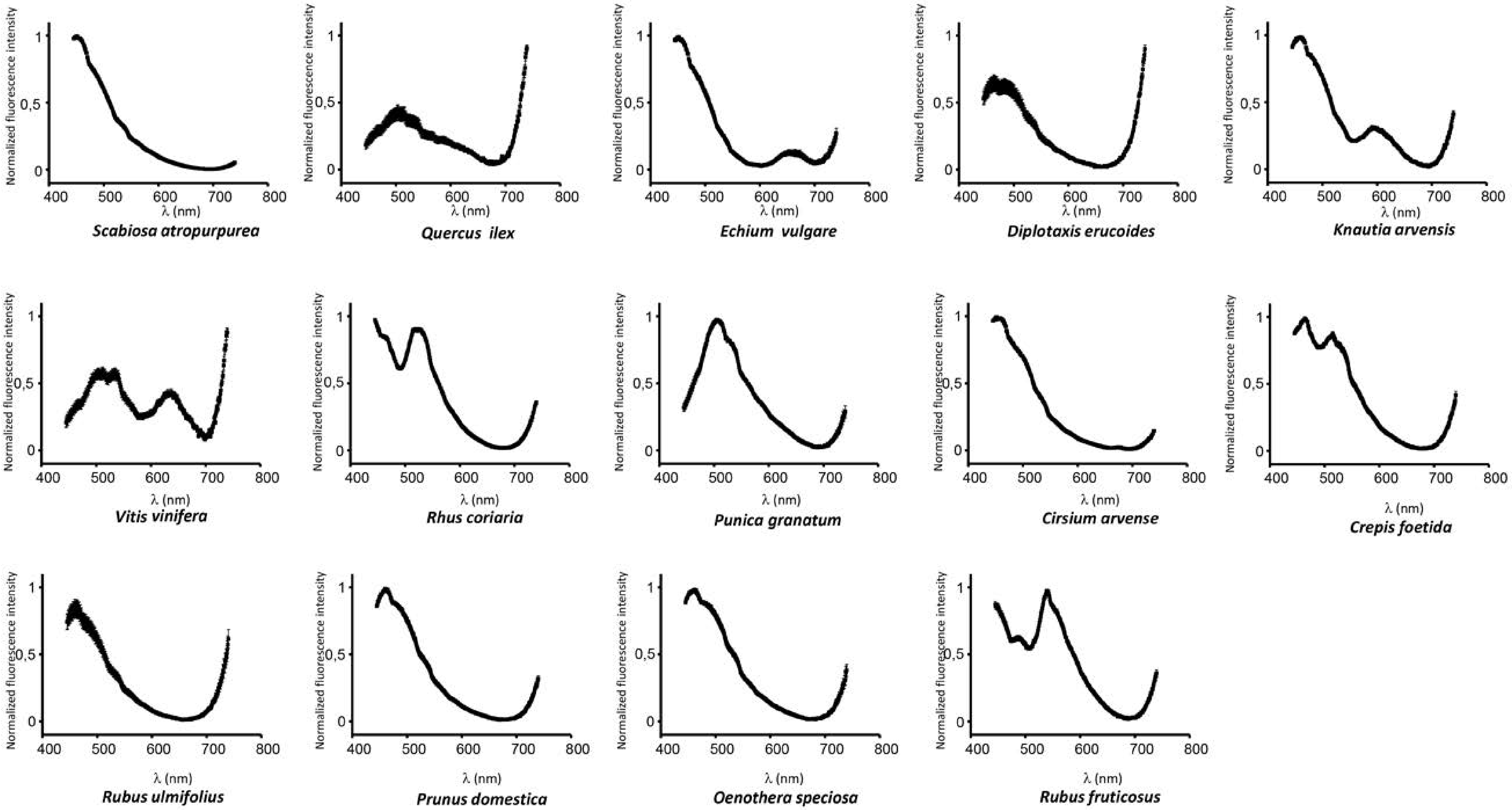

SUPPLEMENTARY FIGURE 4 (Excitation wavelength 460nm)

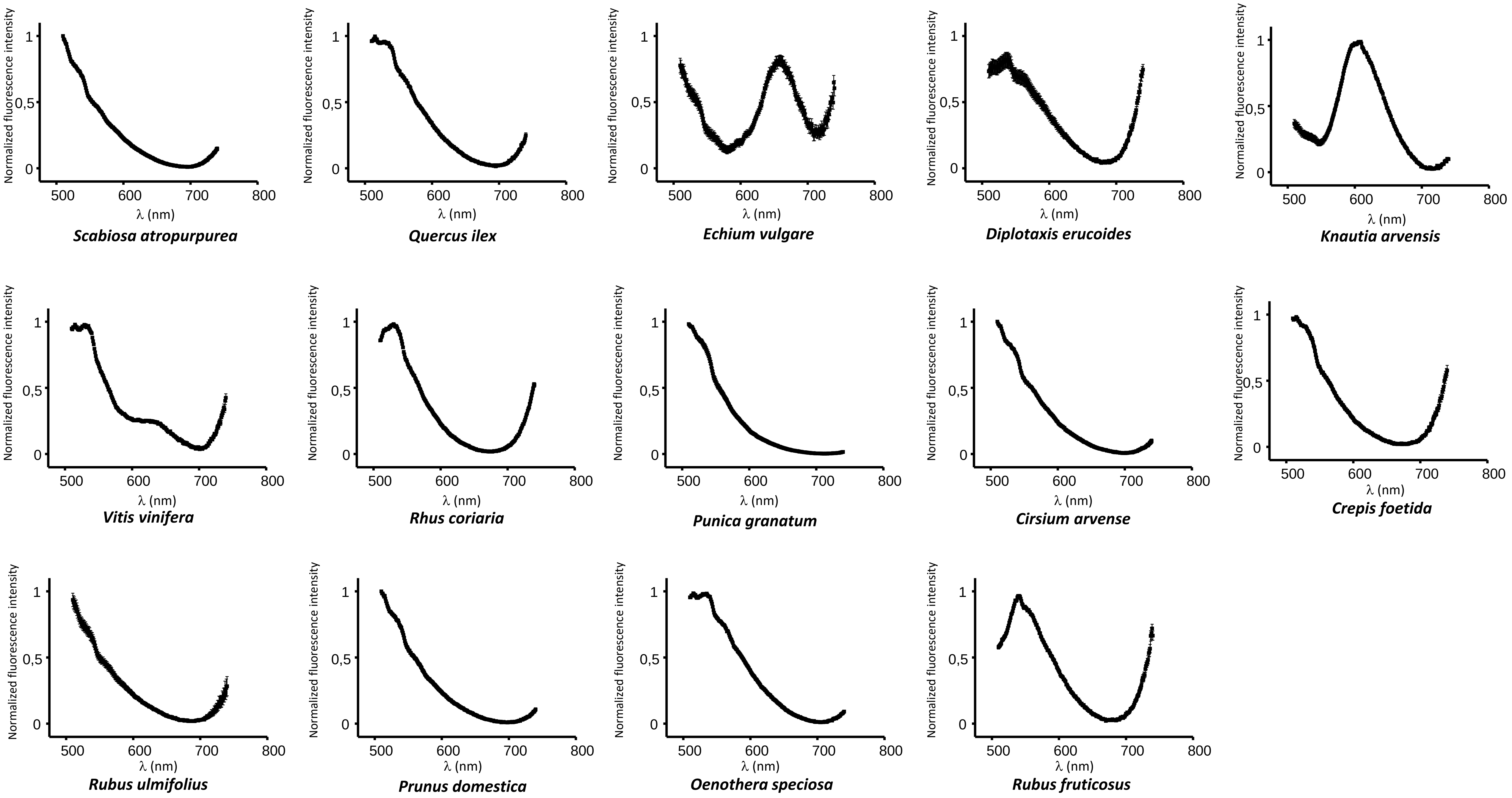

SUPPLEMENTARY FIGURE 5 (Excitation wavelength 530nm)

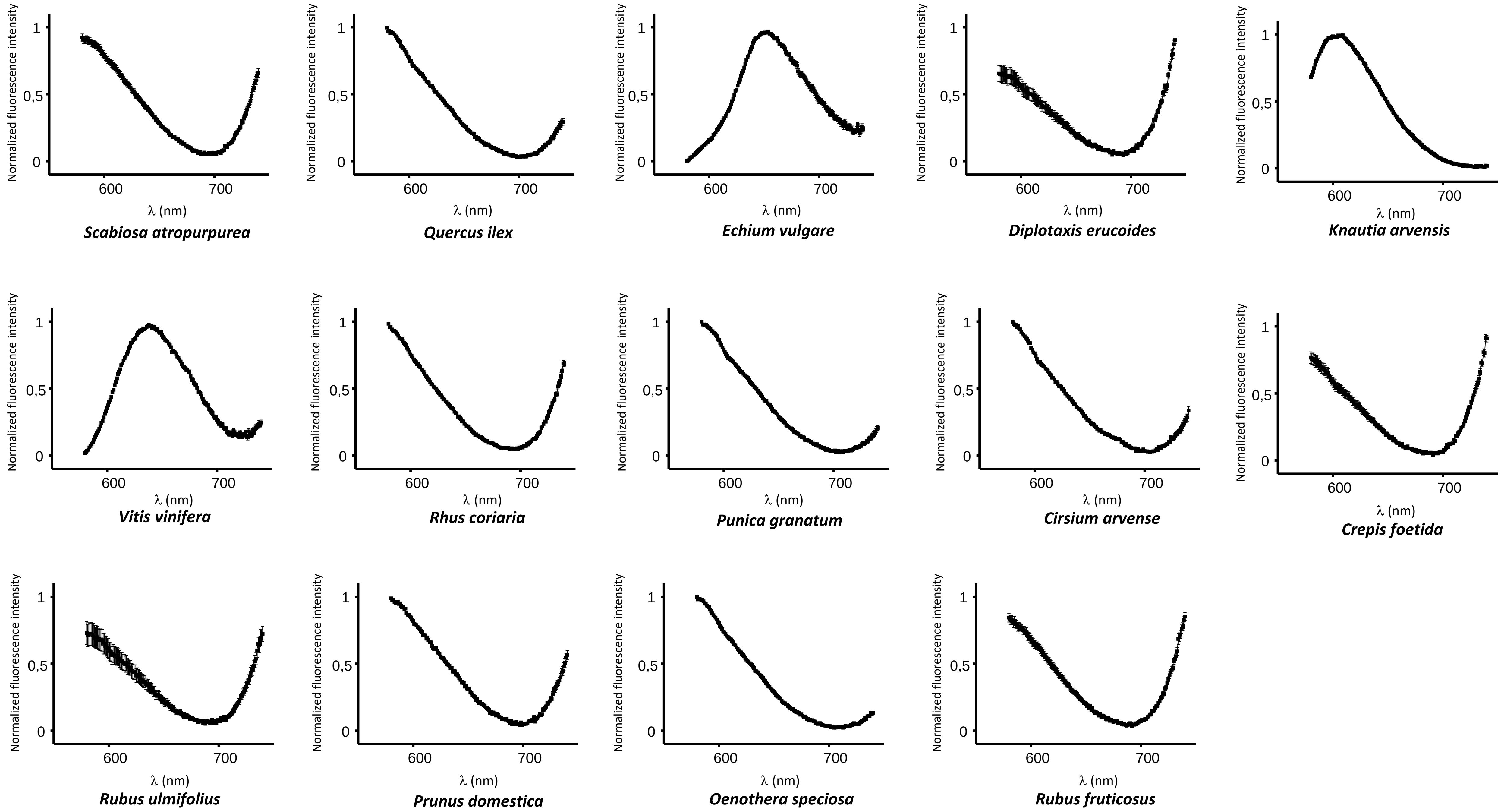

SUPPLEMENTARY FIGURE 6 (Excitation wavelength 625nm)

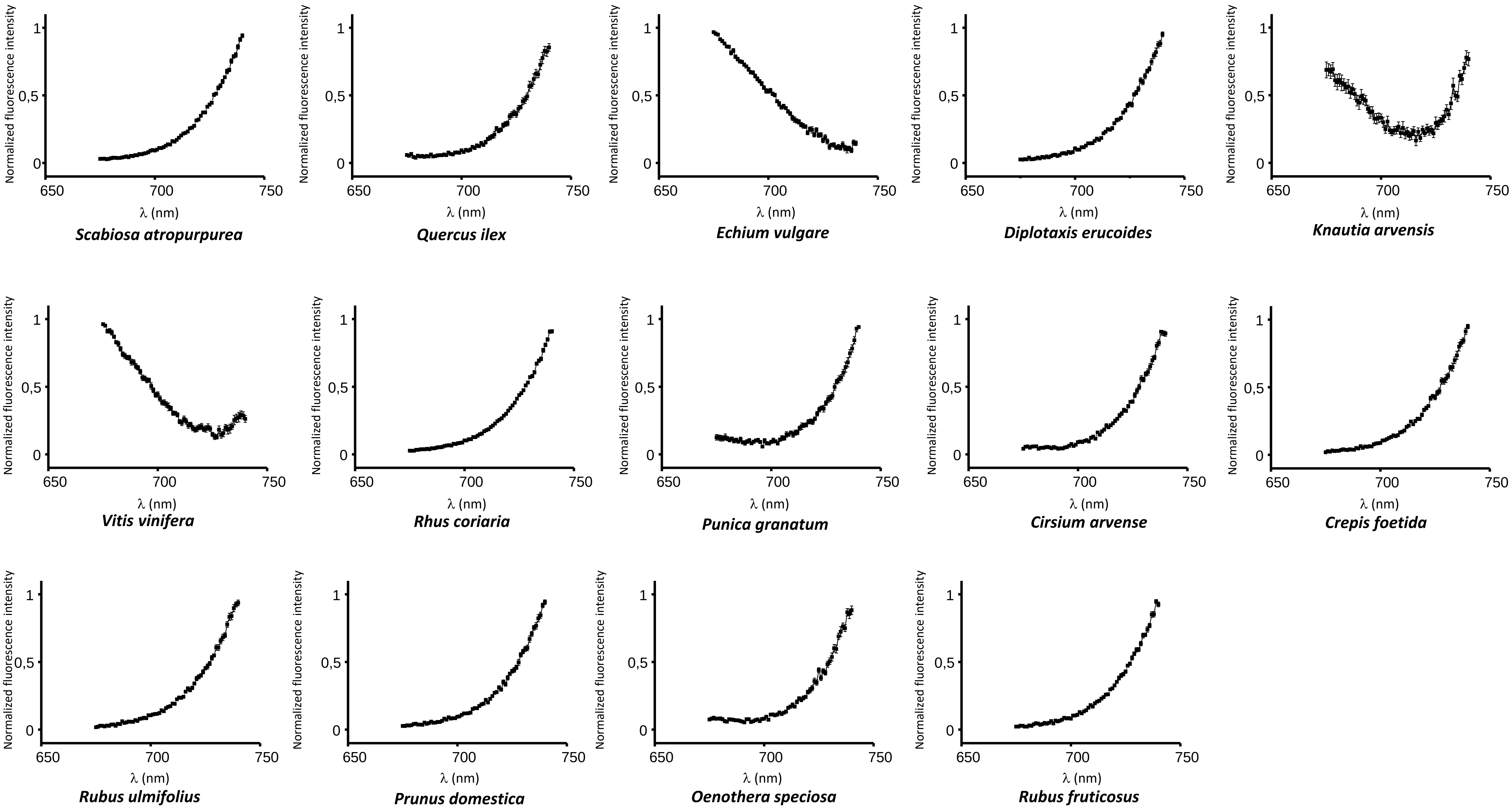

SUPPLEMENTARY FIGURE 7

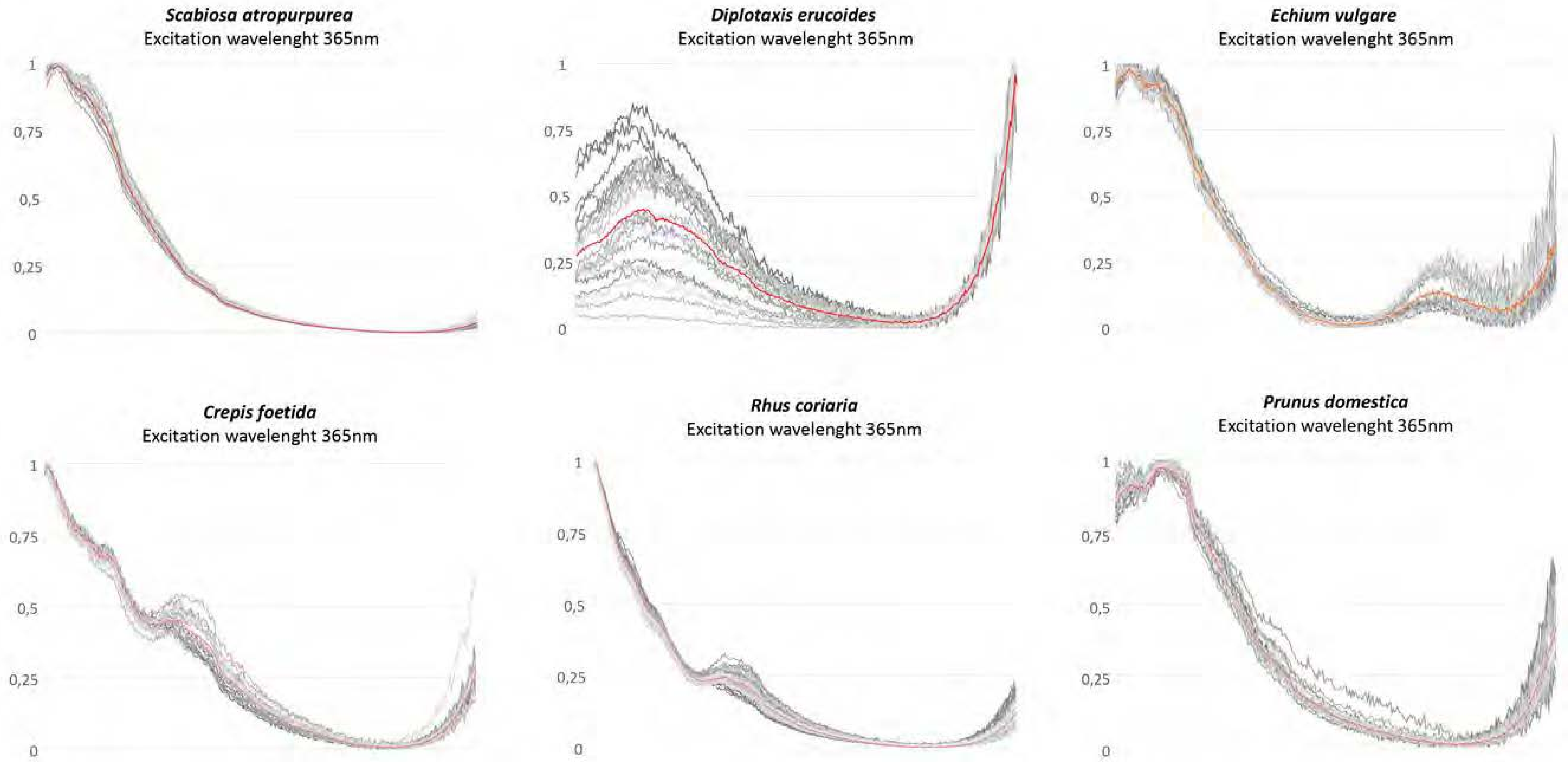

SUPPLEMENTARY FIGURE 8

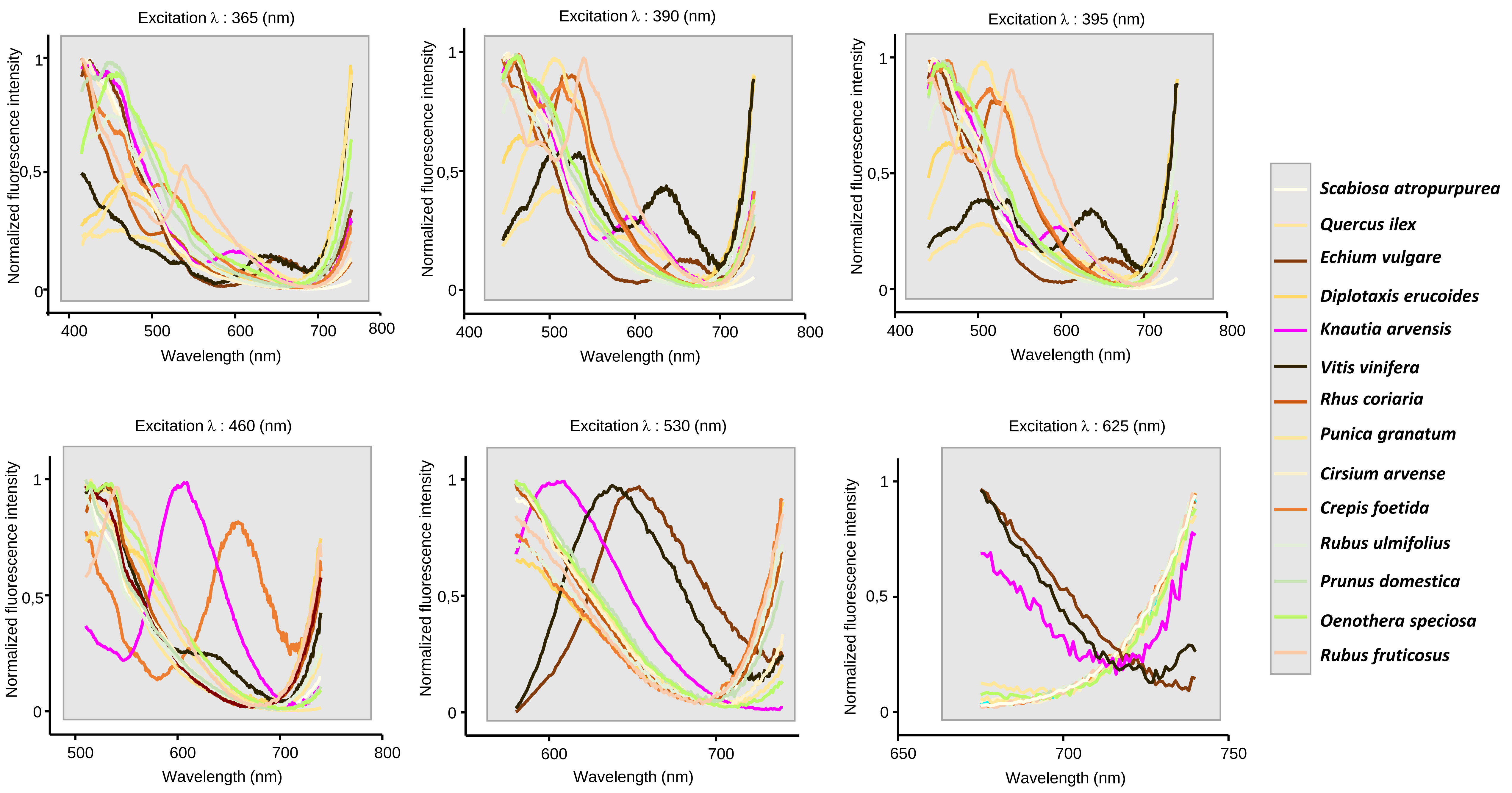

SUPPLEMENTARY FIGURE 9

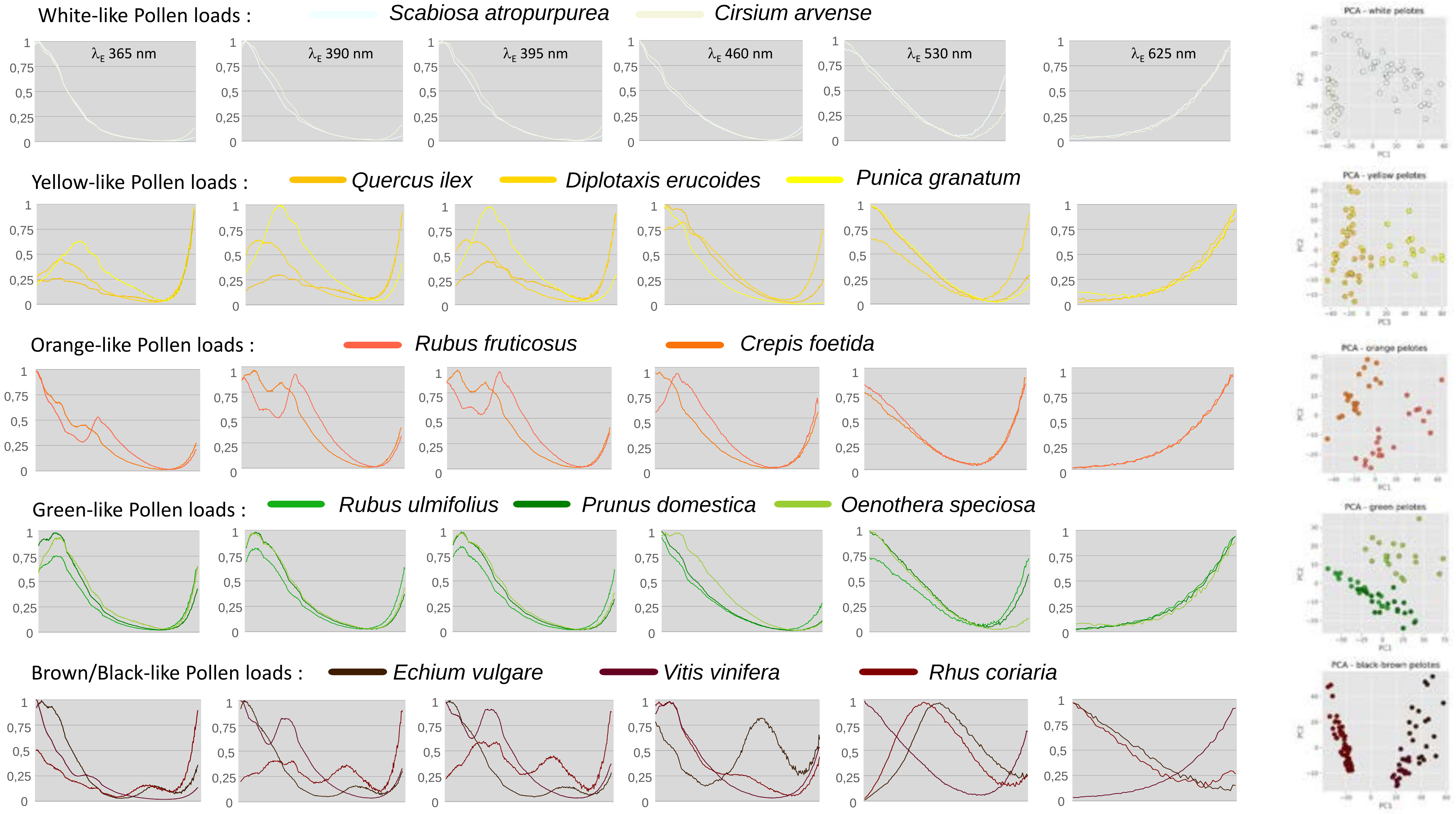

SUPPLEMENTARY FIGURE 10

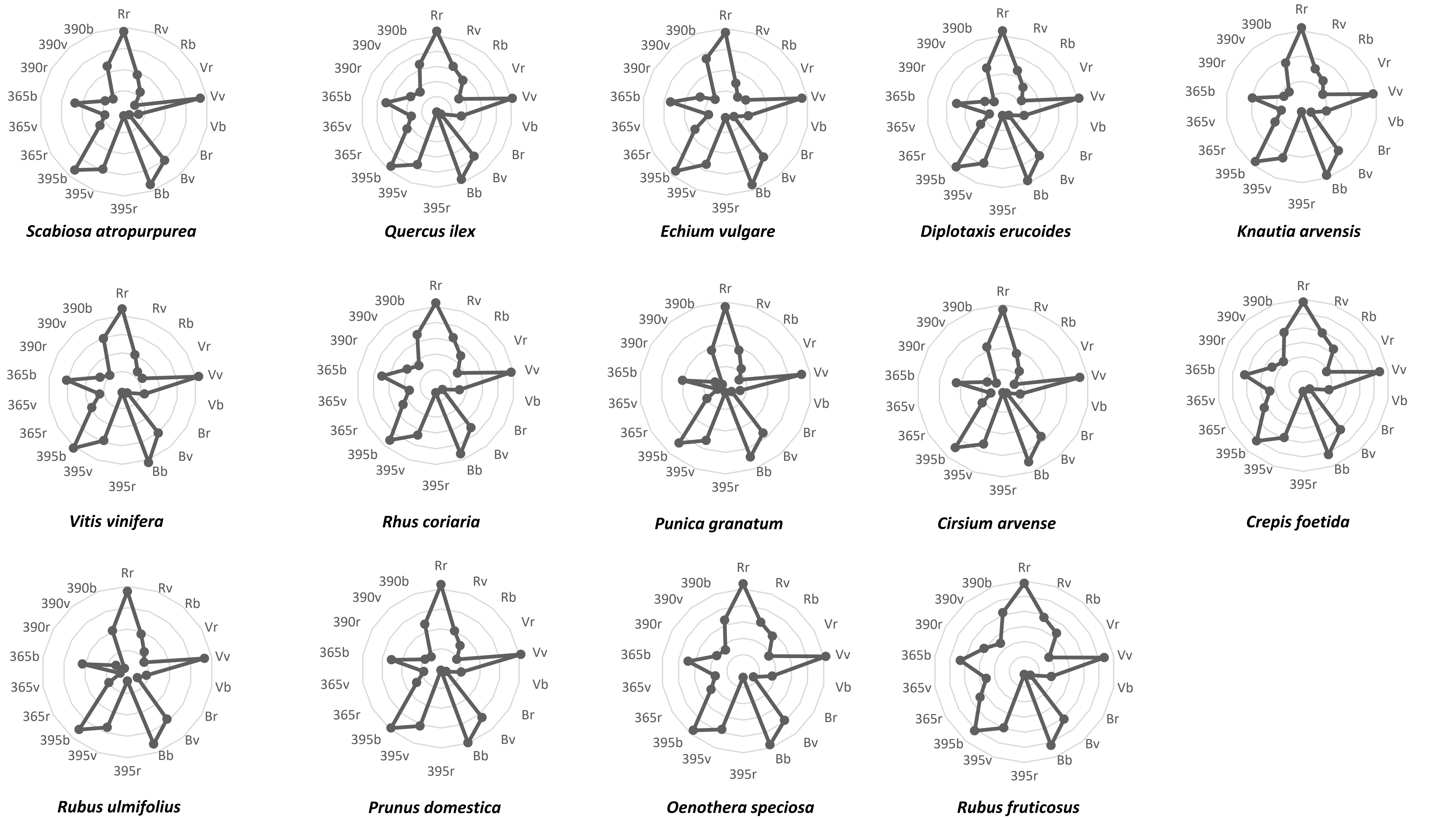

SUPPLEMENTARY FIGURE 11

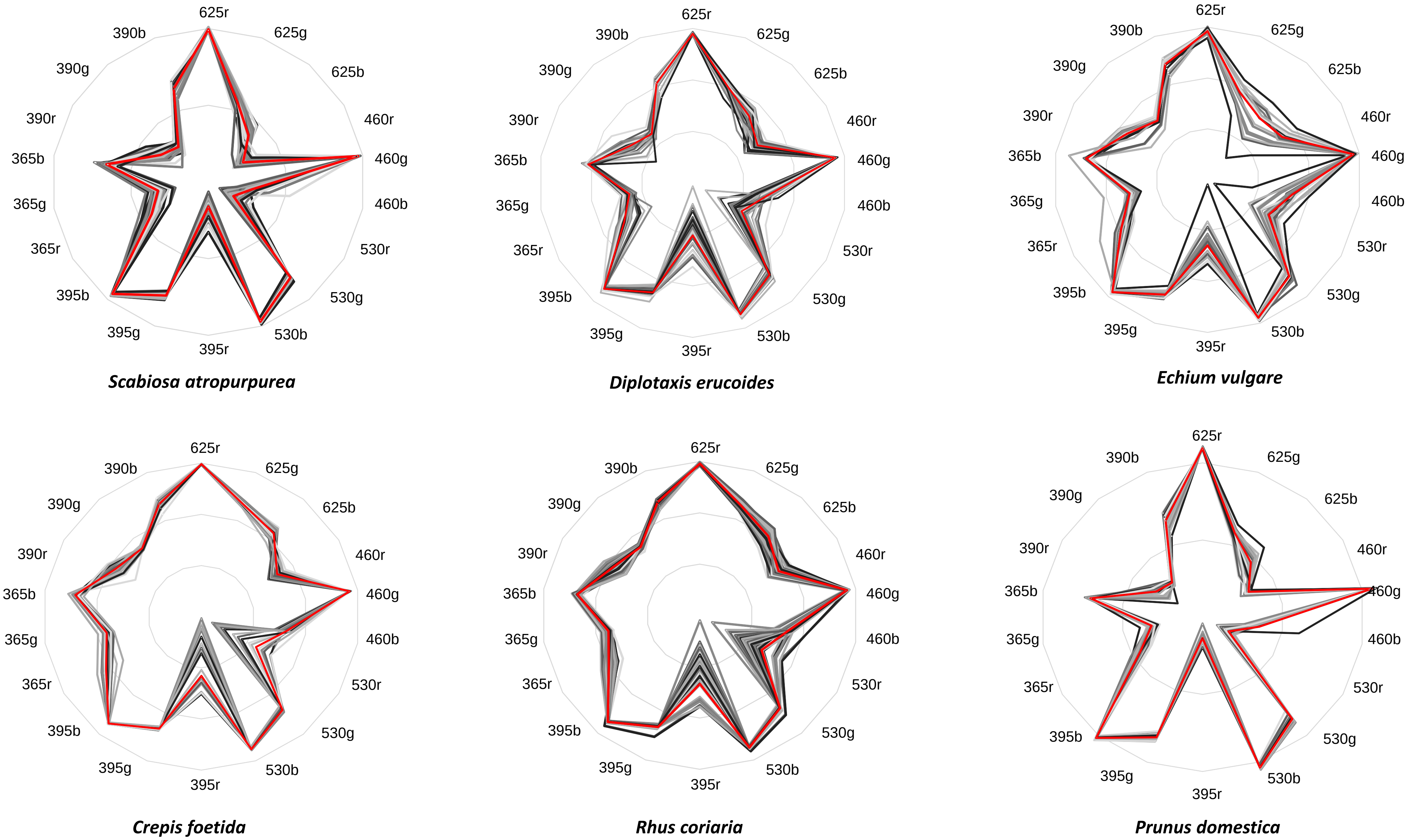

SUPPLEMENTARY FIGURE 12

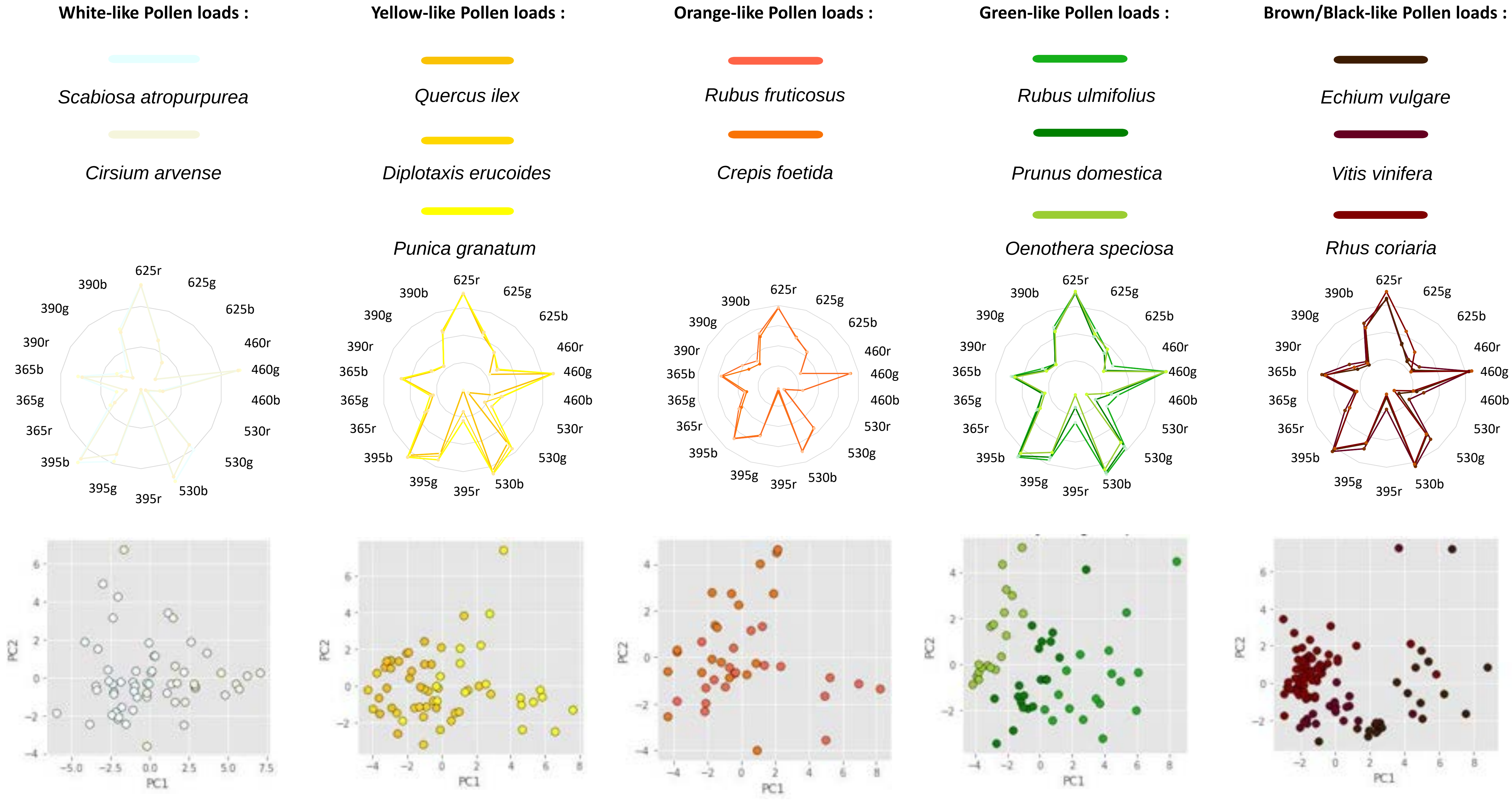

Supplement: Supplementary file 2 — Fig. S1 Normalised average fluorescence responses of the 14 species studied induced by an excitation at 365 nm acquired through fluorescence spectroscopy. Fig. S2 Normalised average fluorescence responses of the 14 species studied induced by an excitation at 390 nm acquired through fluorescence spectroscopy. Fig. S3 Normalised average fluorescence responses of the 14 species studied induced by an excitation at 395 nm acquired through fluorescence spectroscopy. Fig. S4 Normalised average fluorescence responses of the 14 species studied induced by an excitation at 460 nm acquired through fluorescence spectroscopy. Fig. S5 Normalised average fluorescence responses of the 14 species studied induced by an excitation at 530 nm acquired through fluorescence spectroscopy. Fig. S6 Normalised average fluorescence responses of the 14 species studied induced by an excitation at 625 nm acquired through fluorescence spectroscopy. Fig. S7 Normalised fluorescence spectra (in grey shade) acquired through fluorescence spectroscopy and average fluorescence spectra (in red) induced by an excitation at 365 nm for 6 species showing the variability obtained with some species. Fig. S8 Superposition of the average fluorescence spectra acquired through fluorescence spectroscopy of the 14 species studied induced by an excitation wavelength of 365, 390, 395, 460, 530 or 625 nm. Fig. S9 Superposition of the average spectra acquired through fluorescence spectroscopy for each excitatory wavelength of species with pollen load with the same tone. Fig. S10 Radar representation of the average data collected with the pollen analyser for each species. Fig. S11 Superposition of the radar graphs of data acquired with the pollen analyser for each pollen load for a given species (in grey) and the average radar graph for this species (in red). Fig. S12 Superposition of the radar graphs of data acquired with the pollen analyser for different species, grouped by similar pollen load colour tones. Please note: [file NPH-248-1584-s001.pdf]
